# Four-Dimensional Printing of Shape Memory Polymers for Biomedical Applications: Advances in DLP and SLA Manufacturing

**DOI:** 10.3390/polym18010024

**Published:** 2025-12-22

**Authors:** Raj Kumar Pittala, Marc Anthony Torres, Neha Reddy, Sara Swank, Melanie Ecker

**Affiliations:** 1 Department of Biomedical Engineering, University of North Texas, Denton, TX 76203, USA; 2 Texas Academy of Mathematics and Science, University of North Texas, Denton, TX 76203, USA

**Keywords:** shape memory polymers (SMPs), DLP, SLA, 4D-printing, biomedical applications, smart materials

## Abstract

Shape memory polymers (SMPs) represent an innovative class of materials that possess programmed, reversible shape-changing capabilities in response to external stimuli. The recent emergence of SMPs’ advanced manufacturing, specifically 4D printing, has created exceptional opportunities for use in biomedical engineering. This review presents a critical synthesis of the latest advances in the chemistry, biomedical applications, manufacturing strategies, and clinical translation of SMPs, highlighting vat photopolymerization techniques, such as stereolithography (SLA) and digital light processing (DLP). Notably, 4D-printed SMPs can promote spatiotemporally controlled architectures, and applications include minimally invasive implants, dynamic tissue scaffolds, and multifunctional drug delivery. This paper focuses on recent advances in resin design, multi-responsive and nanocomposite resins, AI-guided material discovery, and emerging biocompatible and biodegradable formulations, while outlining current roadblocks to clinical implementation, including cytotoxicity, sterilization, regulatory compliance, and device shelf-life. Our goal is to elucidate the relationship between material design, processing, and biomedical performance to inform researchers of potential future directions for 4D-printed SMPs and next-generation, patient-centered medical devices.

## 1. Introduction

**Figure 1:**
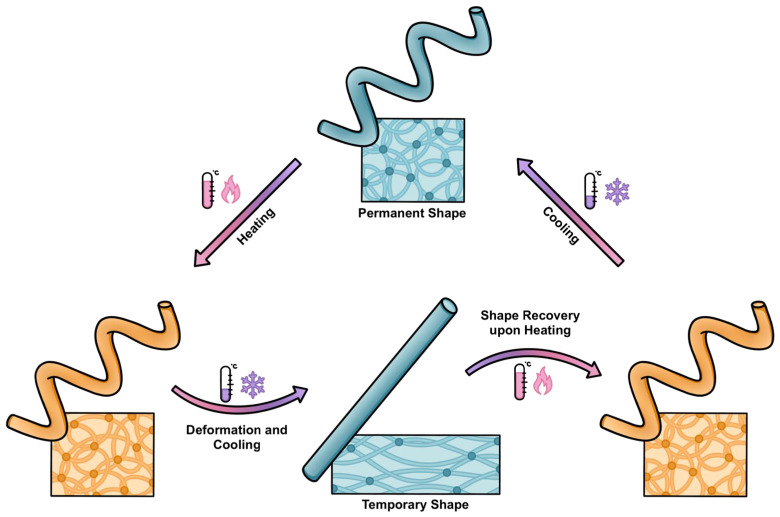
Schematic representation of shape memory effect (SME), in which the material can be temporarily deformed below the transition temperature (*T_trans_*), and returns to the permanent, pre-programmed shape upon subsequent heating.

Shape memory polymers (SMPs) are stimuli-responsive smart materials, which possess a preeminent ability to return to their predetermined permanent shape following mechanical deformation through the application of certain external triggers. This is achieved through induction of shape memory effect (SME), which causes the polymer chains to assume an entropically favorable conformation, varying with chemical composition and morphology [1]. The mechanism utilized during SMP synthesis, either physically or chemically crosslinked, defines the structural properties, which can be physically or chemically crosslinked. Activation of SME (Figure 1) is based on external stimuli, such as temperature, electromagnetic radiation, hydration, and/or chemical cues [2,3,4,5,6]. Most commonly reported in the literature, temperature-induced triggers have been explored for their biocompatibility, tunable mechanical properties, and responsiveness. SMPs are tackling existing biomedical problems with novel solutions, and recent advances in manufacturing techniques, like 4D and vat printing, open new possibilities for customizing functionality with near-limitless control over material design, architecture, and fabrication [7,8,9].

This comprehensive review aims to provide an in-depth analysis of the current state and future prospects of 4D-printed SMPs in biomedical applications, specifically outlining up-to-date FDA regulatory pathways in comparison to existing 3D-printed implantable devices (Class 3 Pre-market Approval (PMA)/De Novo requirements). By examining the interplay between material properties, SME activation mechanisms, compatibility with advanced manufacturing processes and sterilization techniques, biological requirements, and design optimization utilizing artificial intelligence (AI), this work seeks to guide researchers, industry professionals, and clinicians in advancing the field toward successful commercialization of 4D-printed biomedical devices.

Typically, materials maintain mechanical, physical, and chemical properties, regardless of environment. In contrast, SMPs can be programmed to exhibit SME, utilizing physiological signals created by the human body, such as temperature, pH, and moisture, to fabricate self-expanding stents, site-specific drug delivery devices, adaptive and dynamic tissue scaffolds, and smart wound dressings [10].

**Figure 2:**
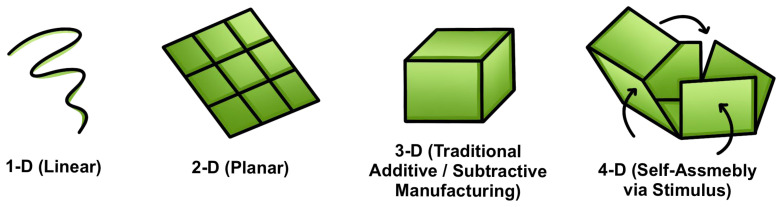
Representation of multi-dimensional geometry, including the addition of temporal programming in 4D-printed constructs.

The shift from traditional SMP fabrication techniques to additive manufacturing, such as 4D-printing, represents a paradigm shift for designing biomedical devices [9]. While traditional solvent casting, electrospinning, and thermomoulding have proven effective in SMP manufacturing, they are limited in terms of their ability to create complex geometries and exert control over spatial material properties [11]. With the development of 4D-printing mechanisms like stereolithography (SLA) and digital light processing (DLP), SMP structures with an unprecedented degree of design flexibly are now possible. Temporal programmability (Figure 2), where printed structures can self-assemble into predetermined conformations after fabrication, is made possible in 4D printing through careful material selection, geometric design, and control of manufacturing parameters that encode the instructions for permanent shape into the printed structure [12,13]. Recent developments in 4D printing include multi-material systems with the ability to generate hierarchically porous structures to replicate natural tissue architectures and the development of resorbable elastomers that produce biocompatible degradation products following cell-mediated tissue remodeling [14].

**Figure 3:**
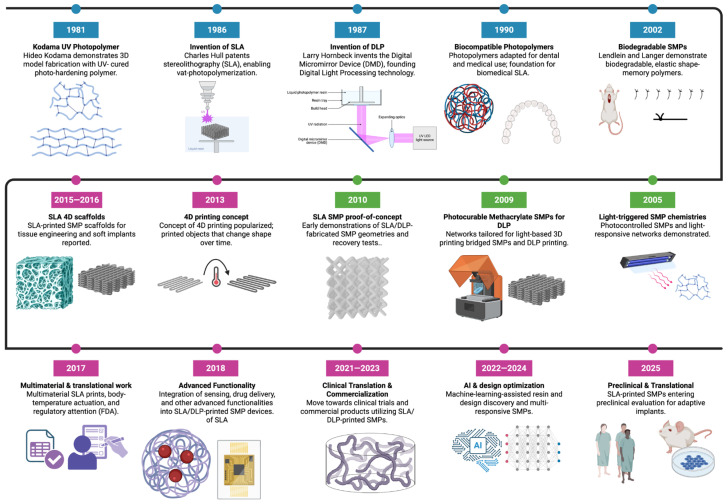
Timeline of the advancement of DLP and SLA printing for biomedical applications.

One of the first landmark developments in light-based additive manufacturing was the conceptualization by Dr. Hideous Kodama in 1981 and the invention of SLA by Chuck Hull in 1986, which enables layer-by-layer fabrication of 3D structures from ultraviolet (UV) photocurable resins using a focused laser (Figure 3) [15]. Although initially applied to rapid prototyping with rigid acrylates, SLA was soon adapted for biomedical use in the 1990s for surgical planning models and dental restorations. Shortly thereafter (late 1980s), Texas Instruments introduced DLP printing, which employs a digital micromirror device (DMD) for 2D projection-based curing as an alternative with faster build speeds and higher throughput compared to point-by-point SLA [16]. Beginning in 2009, Yackaki and collaborators utilized existing photopolymerization systems to fabricate methacrylate-based SMP networks, which included remote heating using Fe_3_O_4_ nanoparticle agents, and tunable glass transition temperatures for suitability in biomedical devices [17].

Novel 4D-printed SMPs for biomedical applications primarily focus material development, improved manufacturing processes, design optimization with AI, and clinical implementation [7,18].

## 2. Fundamentals of Shape Memory Polymers

### 2.1. Molecular Mechanisms

Shape memory effect is not an intrinsic property of the polymers, requiring novel processing steps informed by a fundamental understanding of functionalization based on entropic elasticity [19]. The internal architecture consists of both net-points and molecular switches associated with the conformational mechanism. The net-points are rigid physical or chemical crosslinks, determining the permanent shape of the material and the overall stability of the polymer network architecture. The molecular switches are stimuli-sensitive segments that allow temporary shape freezing and subsequent shape recovery. These molecular switches can be amorphous or crystalline regions within the polymer network, fixing the mechanically deformed shape through additional reversible crosslinks [20]. The moieties with the highest thermal transition temperature (*T_perm_*) act as net-points, while the segments with second-highest thermal transition temperature (*T_trans_*) act as molecular switches [19].

The shape memory effect can be thermodynamically explained with entropic elasticity. In their permanent shape, polymeric chains possess high entropy in a randomly coiled state. When the polymer system is mechanically deformed into its temporary shape below *T_trans_*, the chains become aligned and stretched, reducing the system’s entropy in a higher energy configuration. Following the second law of thermodynamics, the system seeks to return to the original, randomly coiled configuration upon activation by an external stimulus to overcome the energy barrier imposed by temporary stabilizing forces [1].

SMPs can lose their conformational flexibility through various mechanisms, such as environmental factors, repetitive mechanical stress, and the inherent nature of the polymer network architecture. Thermal degradation, caused by continuous exposure to temperatures exceeding the material stability limit, can break down the network, which is crucial for polymer shape memory behavior [1]. Mechanical fatigue from cyclic loading and unloading, especially at high strains, can cause damage, thereby reducing its ability to store and release elastic energy. Environmental factors, such as moisture, UV radiation, or chemical exposure, can degrade the polymer over time [21]. Improper fabrication procedures, like inadequate stress-relaxation time or inappropriate programming temperatures, can lead to poor shape fixity and shape recovery of the polymer [22].

### 2.2. Classification of SMPs

SMPs are differentiated by the following: (1) network architecture, including physically crosslinked (e.g., with freely mobile chains held together by reversible noncovalent junctions, such as hydrogen bonds or crystallites), chemically crosslinked (e.g., with permanent covalent bonds), and hybrid networks (e.g., a dual polymer system that combines a physical and chemical network to afford strength and possibility of reprogramming) [23]; (2) actuation mechanisms, including heating (e.g., thermal glass or melting transitions) [24], water (plasticization with increased chain mobility), light (photochemical crosslinks or photothermal heating), electric or magnetic field (either resistive heating or inductive heating), ultrasound [3], and/or pH (to create site-specific activation); and (3) behavior in cycles, including one-way SMPs (require reprogramming for each cycle, but offer high fixity and recovery) and reversible two-way SMPs (enable shape change in both directions and afford the ability to do many shape changes without having to reset each time, with an application in biomimetic actuators and self-adjusting devices). These different types of systems allow for a rational approach to the design of shape memory polymers for a range of applications.

Physical crosslinks: Permanent network structures can be established in SMPs via physical crosslinks in the form of noncovalent interactions, such as hydrogen bonds, crystallites, or microphase-separated domains. Within molecular switches, physical crosslinks temporarily anchor polymer chains in place after deformation. Upon heating above the transition temperature (glass transition *T_g_* or melting *T_m_*) or light activation where the physical crosslinking is disrupted, the network reorganizes and relaxes back, thereby recovering the shape. Therefore, physical crosslinked SMPs may provide easy reprocessing and recycling, but are typically accompanied by lower shape-fixity ratios (*R_f_*) and generally weaker mechanical properties compared to covalent SMPs [23].

Chemical crosslinks: Chemically crosslinked SMPs use permanent covalent bonds. These are created using multifunctional monomers, such as diacrylates and diisocyanates, to create a permanent 3D network. This structure provides high shape-fixity (*R_f_* > 95%) and nearly full shape recovery (*R_r_* > 98%) for many cycles with enhanced performance in the presence of mechanical load and environmental stimuli. Chemically crosslinked SMPs are typically thermally actuated, which makes them ideal for applications requiring precision, such as in stents, but they cannot be reprocessed and are limited in terms of recycling capabilities [23,25].

Hybrid: Hybrid SMPs employ a combination of permanent covalent crosslinks and reversible physical intermolecular forces, allowing for interpenetrating network architectures. The permanent (covalent) network provides mechanical robustness and stability, while the physical (reversible) crosslinks facilitate dynamic reprogramming, multi-stimuli response, and possibly sequential multi-shape transitions. Hybrid SMPs are synthesized so that the proportions of chemical and physical crosslinks achieve stability, while still allowing for structural reversible adaptability, and both properties are needed for smart devices that are designed to impart reliable support and customizable activation [25].

Thermoresponsive: Thermoresponsive SMPs are still the most studied class, where either *T_g_* or *T_m_* is exceeded to trigger shape recovery. Programming is achieved by manufacturing at relatively high temperature, and subsequent deformation to entropically unfavorable state, which is subsequently fixed upon cooling below *T_g_* or *T_m_*. When reheated, the permanent shape is recovered. These SMPs are often the basis for 4D-printed biomedical scaffolds [2,24,26,27].

Water-activated: Water-activated SMPs use plasticization to decrease *T_g_* below environmental temperature when immersed in water or bodily fluids. This enables indirect thermal activation to deploy minimally invasive, self-expanding stents and embolic foams. Importantly, water-activated SMPs require physiological environments to trigger in vivo deployment and/or drug delivery, and will not exhibit SME in dry storage [2,5,28].

Photoresponsive: Photoresponsive SMPs utilize reversible photochemical crosslinks (such as cinnamate or anthracene dimerization) or photothermal additives (e.g., Au nanorods, CNTs, polydopamine) for actuation controlled by light. When these SMPs are irradiated at specific wavelengths, the energy applied can either break or form crosslinks (e.g., UV), or convert light into heat (e.g., NIR), allowing for remote, spatiotemporal recovery in biomedical and microelectronics devices [29,30,31].

In addition to heat, hydration, and light, SMPs have also been activated by magnetic fields [32] (e.g., magnetic nanoparticle-induced inductive heating), electric currents (e.g., resistive heating in conductive composites), ultrasound [3] (e.g., acoustic heating), and pH changes (e.g., ionic crosslink modification). These alternatives expand the toolkit for remotely triggered, site-specific activation.

There are ongoing developments in two-way, or reversible shape memory polymers, which are able to switch between two different shapes cyclically without reprogramming. Mechanisms range from crystallization-induced elongation and a melting-induced contraction for constant load, or via bidirectional phase transitions in dual-*T_m_* networks. Reversible shape memory polymers provide applications in artificial muscles, dynamic implants, and adaptive sensors in which actuation is cyclic.

### 2.3. Quantification of Shape Memory Effect

Quantitative characterization of SME is investigated using specific protocols in dynamic mechanical analysis (DMA) or universal tensile testing (UTT) machines equipped with a thermo-chamber for cyclic thermomechanical testing [33,34].

There are three key parameters that can quantify the SME of the polymer system: shape fixity ratio (*R_f_*) measures the ability of the material to maintain its temporary shape strain after unloading (*ε_u_*) based on the maximum strain applied during deformation (*ε_m_*). Here, *ε_u_*(*N*) is the strain measured after unloading in the *N*th cycle.

(1) $$R_{f}(N)=\frac{\varepsilon_{u}(N)-\varepsilon_{p}(N-1)}{\varepsilon_{m}-\varepsilon_{p}(N-1)}$$

Shape recovery ratio (*R_r_*) quantifies the material’s ability to return to its original shape, based on the permanent strain after recovery. *R_r_* is typically evaluated per-cycle and compared to the previous cycle, where (*ε_m_*) is the maximum strain applied to the sample *ε_p_*(*N*) is the permanent strain remaining after *N* cycles.

(2) $$R_{r}(N)=\frac{\varepsilon_{m}-\varepsilon_{p}(N)}{\varepsilon_{m}-\varepsilon_{p}(N-1)}$$

Total shape recovery ratio (*R*_*r*,*tot*_) defines total strain recovery, and the ability of the material to return to its original geometry after *N* cycles.

(3) $$R_{r,tot}(N)=\frac{\varepsilon_{m}-\varepsilon_{p}(N)}{\varepsilon_{m}}$$

The above calculations are typically performed for multiple cycles to assess the consistency for repeated use. By analyzing these ratios across different temperatures and multiple cycles, the shape memory effect can be characterized to evaluate short-term and long-term performance, allowing for optimization of material composition and processing conditions to achieve desired shape memory properties for specific applications.

## 3. Advanced Manufacturing Techniques of Shape Memory Polymers

Additive manufacturing (AM) has emerged as a powerful tool for creating customized 3D structures, with broad applications in biomedical engineering, such as microfluidics, tissue engineering, and wearable sensors [35,36,37]. A wide range of AM techniques have been developed, including liquid crystal display (LCD) 3D-printing, fused deposition modeling (FDM), selective laser sintering (SLS), and directed energy deposition (DED) [38,39]. This review focuses primarily on SLA and DLP, which can be used in the 4D-printing of SMPs [40].

**Figure 4:**
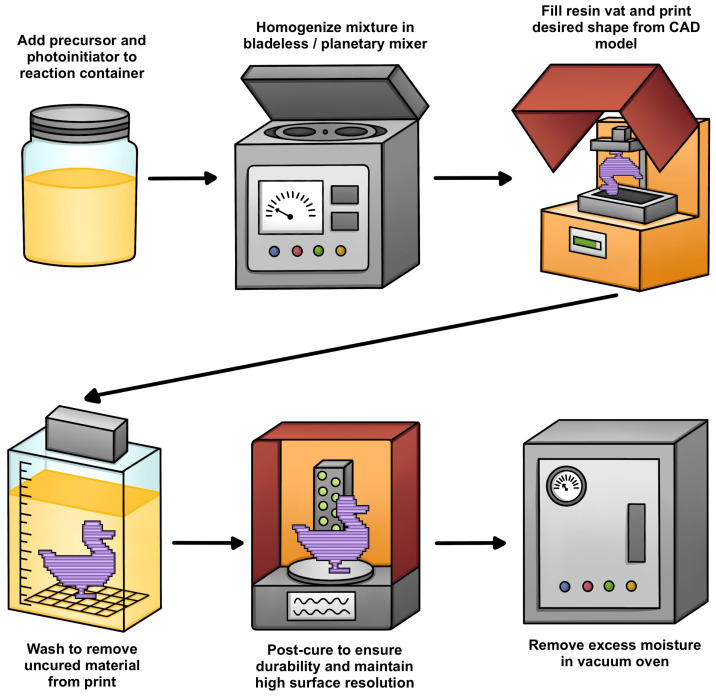
General workflow of SLA/DLP additive manufacturing process.

Thermoset SMPs, which are chemically resistant and possess superior *T_g_* and storage moduli compared to their thermoplastic counterparts, are usually utilized in biomedical applications [41]. Fabrication of thermoset SMPs typically occurs via vat photopolymerization, using UV or visible light to trigger radical-mediated or Michael-type reactions. Photocurable resin formulations commonly include a prepolymer (oligomer) and a photoinitiator (Figure 4).

### 3.1. Stereolithography (SLA)

**Figure 5:**
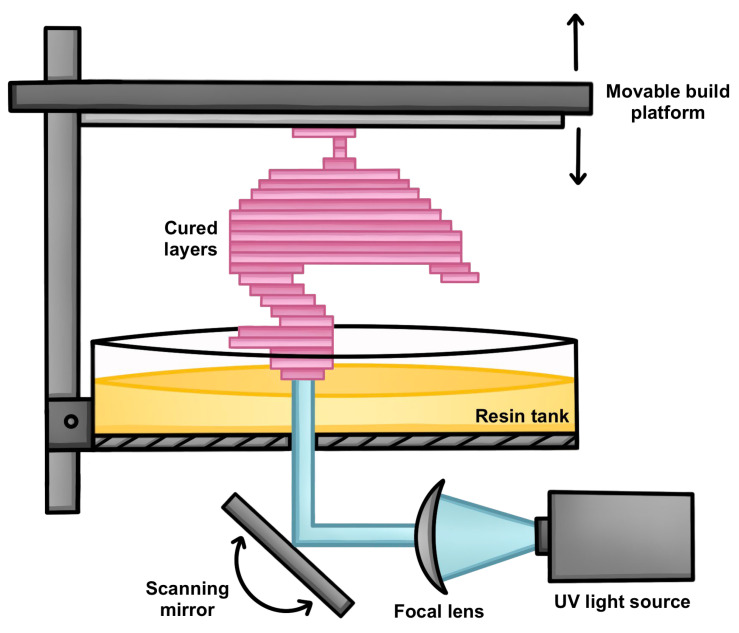
Schematic of SLA printing process.

In practice, SLA printing (Figure 5) begins with a vat of liquid resin (oligomers and liquid monomers) that is selectively solidified by UV exposure, with photoinitiators (PIs) in the resin formulation acting as catalysts to trigger localized photopolymerization upon irradiation. The UV-exposed regions then undergo a curing reaction involving two key transitions: gelation and vitrification. Gelation marks the transition from a liquid to a rubber-like state through increased viscosity, while vitrification represents a shift to a rigid, glassy solid. Notably, vitrification is both incremental and thermo-reversible, enabling greater control over the material’s final properties [42].

Typically, acrylate-based resins are highly favored in SLA printing due to their high reactivity and rapid building speed. Their thermal resistance and mechanical properties can be finely tuned by modifying the number of reactive groups, making them particularly suitable for SLA applications. Across the studies mentioned in this section, it is important to highlight that polyurethane-based SMPs slightly outperformed acrylate-based SMPs in shape memory performance metrics, exhibiting shape fixity (*R_f_*) values exceeding 96% and nearly perfect shape recovery (*R_r_*) values (~100%).

In a study by T. Zhao et al. [43], a modified polyurethane acrylate (PUA) was synthesized and combined with isobornyl acrylate (IBOA), epoxy acrylate, and a radical photo-initiator (PI 184) to formulate a photosensitive resin for fabricating SMPs via SLA. The study aimed to develop a simple, yet effective method for printing complex thermoplastic shape memory polyurethane (SMPU) structures by evaluating print accuracy. Results showed that the polymer system exhibited high photopolymerization activity and deep UV penetration due to its optical transparency. The printed objects displayed excellent dimensional accuracy, with deviations under 190 µm even at high scanning speeds of 4000 mm/s [43].

**Figure 6:**
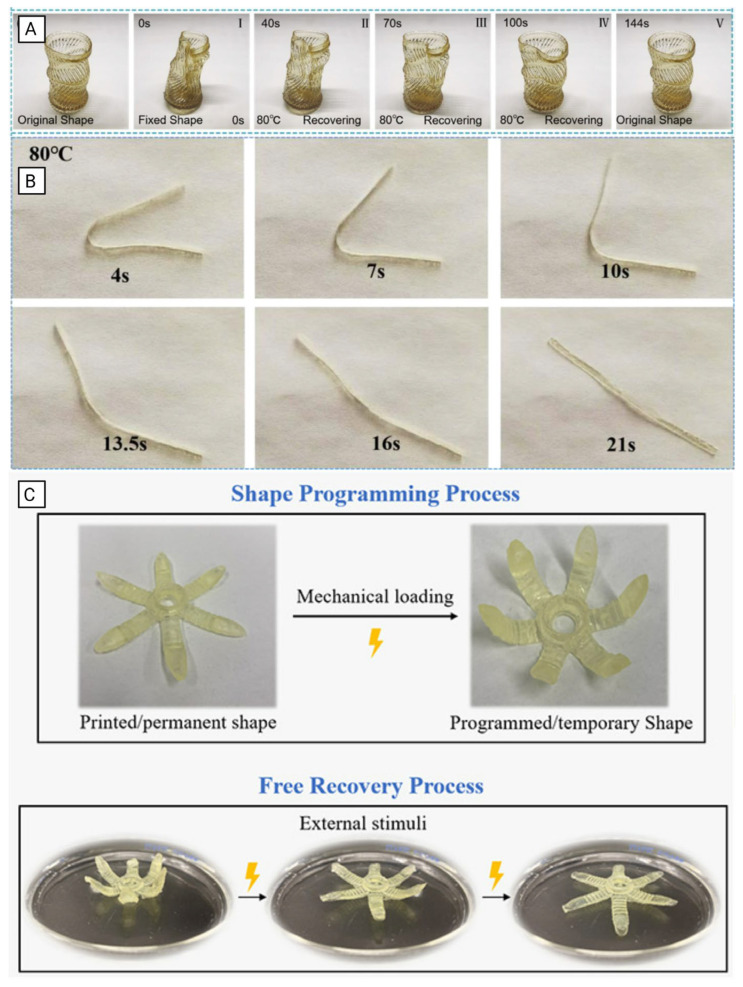
(**A**) Shape recovery process of an SMP pencil holder comprised of an epoxy soybean oil-based composite embedded with itaconic acid monomethyl ester (IAME) modified cellulose nanocrystals (CNCs-IAME). I–V represents the shape memory cycle. Reprinted from Ref. [44]. (**B**) Shape memory recovery process of an epoxy acrylate SMP in 80 °C temperature. Reprinted from Ref. [39]. (**C**) Shape programming and recovery in 4D printing. The flash symbol represents the stimuli. Reprinted from Ref. [45].

To assess shape memory performance, fold-deploy tests were conducted during shape memory cycles (SMCs), visualized in Figure 6B. These tests revealed a strong temperature dependence of chain mobility in crosslinked polyurethane networks, with recovery times decreasing from 18 s at 75 °C to 8.5 s at 90 °C. The DMA of the samples confirmed a strong shape memory behavior, with an elastic modulus ratio (*E*′*_g_*/*E*′*_r_*) of 253.

While high crosslinking densities endowed the SMPUs with outstanding average *R_f_* and *R_r_* ratios (*R_f_* = 96.77 ± 0.06%; *R_r_* = 100.00 ± 0.08%) after 16 cycles, as well as strong tensile and flexural strengths of 37.2 MPa and 49.5 MPa, respectively, this system is still flawed. Specifically, the SMPU structures require abnormally high activation temperatures, making them unsuitable for biomedical applications.

In a study conducted by J. Zhao et al. [45], thermo-responsive methacrylate copolymers were subjected to iterative thermomechanical loading to analytically characterize and experimentally validate their cyclic shape memory effects. The polymer network was composed of three key chemical constituents: benzyl methacrylate (BMA) as a linear chain builder, poly(ethylene glycol) dimethacrylate (PEGDMA) as a crosslinker, and phenylbis(2,4,6-trimethyl benzoyl) phosphine oxide (BAPO) as a photoinitiator. Researchers fabricated various test specimens, including a gripper illustrated in Figure 6C, to evaluate both printability and shape memory capabilities [45].

The printed gripper seen in Figure 6C was subjected to a full thermomechanical cycle to demonstrate shape programming and recovery. In addition, computational model predictions of shape memory behavior were compared to experimental results to ensure accuracy. Across thermomechanical cycles, these SMPs exhibited an average free recovery of 95.73% and a shape fixity of 96.24%, confirming the model’s predictive reliability. Unlike the polyurethane-based SMPs, the emphasis in this work was centered around modeling accuracy rather than mechanical reinforcement.

### 3.2. Digital Light Processing (DLP)

**Figure 7:**
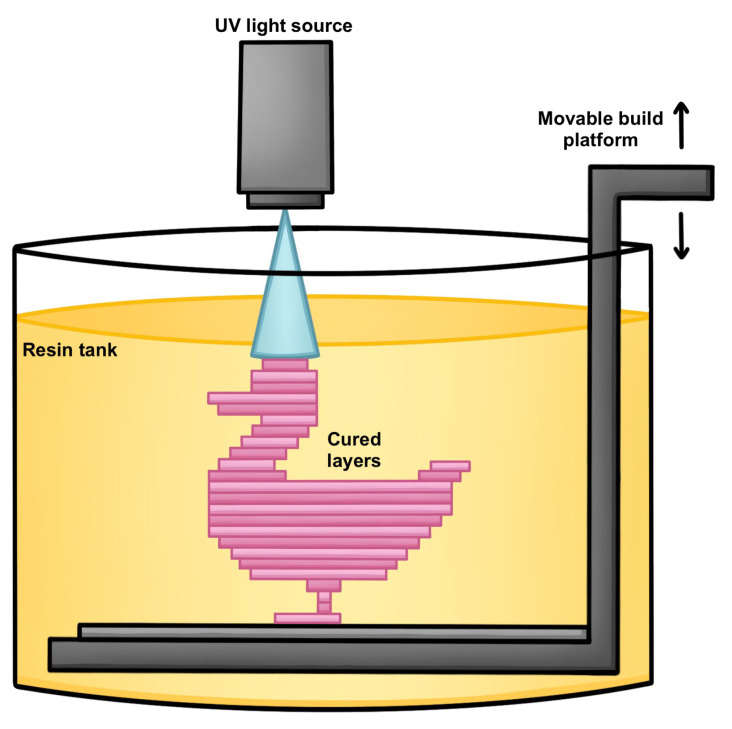
Schematic of DLP printing process.

DLP is a vat photopolymerization-based 3D-printing technique that enables rapid, high-resolution fabrication of both micro- and macro-scale structures [46]. It operates by projecting 2D light patterns onto a vat of photocurable resin using a digital projector, initiating localized photopolymerization. This process is repeated layer-by-layer, resulting in the formation of a final 3D object (Figure 7). DLP printing offers several advantages, including minimal use of support materials, efficient printing of suspended features, and high production throughput. A wide variety of materials can be formulated into printable resins for this technique, such as ceramics, metals, ionogels, elastomers, rigid polymers, and hydrogels.

**Figure 8:**
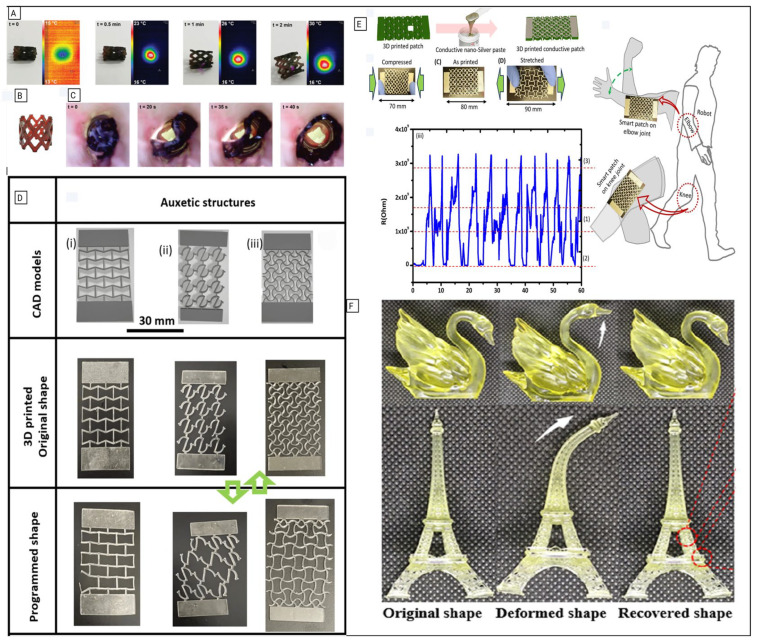
(**A**) Optical and thermal images of meshed DLP-printed SMP stent upon exposure to 808nm laser at different time points. Reprinted from Ref. [47]. (**B**) 3D-printed SMP composite stent. Reprinted from Ref. [47]. (**C**) Programming and recovery in ex vivo porcine intestinal segment for NIR light irradiation. Reprinted from Ref. [47]. (**D**) Original and printed shapes of lattice structures. (i)–(iii) represents the different CAD models. Reprinted from Ref. [48]. (**E**) Application of 3D-printed smart structure for the joint movement sensing application. Reprinted from Ref. [48]. (**F**) DLP 3D printing display of swan and Eiffel tower constructs, composed of poly(glycerol sebacate) acrylate-co-hydroxyethyl methacrylate (PGSA-co-HEMA). Reprinted from Ref. [49].

The adaptability of the resins used in DLP printing enables precise tuning of mechanical properties, ranging from stiff, durable materials to soft elastomers that mimic biological tissues. Leveraging this versatility, Paunović et al. developed bioinks incorporating functional polymers and gold nanorods (AuNRs), which exhibited both elastomeric behavior and strong responsiveness to near-infrared (NIR) light, enabling photothermal actuation [47]. The optimized resins demonstrated high efficiency and structural reproducibility, and printed constructs were shown to degrade in physiological conditions in both ex vivo and in vitro models. Using these DLP-formulated inks, NIR-responsive SMP stents, such as the one shown in Figure 8B, were fabricated, with variations in photopolymer composition. Integration of AuNRs into 4D structures allowed for thermally induced shape change, enabling the SMPs to expand upon heating due to light exposure, as presented in Figure 8A, ultimately intended to facilitate minimally invasive deployment. In application, Figure 8C demonstrates the stent’s deployment in a porcine intestinal section and subsequent rapid, 40 s recovery in response to NIR light.

Despite inclusion of AuNRs, the polymer system is not burdened by severe stiffening, evident by a measured tensile stress of 4.3 MPa, an elongation at break of 124%, and excellent elasticity, as indicated by a Young’s modulus of 4.0 MPa. Importantly, exposure to NIR light did not compromise the mechanical performance of the polymer matrix.

Alam et al. [48] reported a facile and rapid method for 3D DLP-printing of a customized resin combining an acrylic-based polymer with liquid crystal (LC, RM257), which introduced thermoresponsive shape memory properties into the printed structures, while simplifying resin preparation. The study focused on tuning mechanical properties through the fabrication of lattice structures instead of relying solely on chemical modification, as shown in Figure 8D [48].

Tensile testing showed that the mechanical properties of the 3D-printed lattices could be tailored by programming the structures into two states: stretched and compressed. Samples fixed in the stretched state exhibited a higher modulus due to stretch-dominated behavior, while compressed-state samples displayed lower moduli but greater toughness, strength, and strain to failure, attributed to bending-dominated behavior.

Given the flexibility of the 3D-printed lattice, strain-sensing tests were performed to assess their application as smart patches for joint-movement monitoring. The nanosilver-coated lattice served as a conductive electrode, with electrical resistance changes measured during stretching and compression associated with gait (Figure 8E) [49].

Tang et al. [50] developed a cyanate ester-based shape-memory interpenetrating polymer network (IPN) suitable for both direct ink writing (DIW) and DLP 3D-printing. The resulting SMP demonstrated low volume contraction, high printing resolution, and outstanding mechanical properties, including a tensile strength of 70 MPa, a high modulus, and a transition temperature (*T_trans_*) near 170 °C. It exhibited excellent shape memory behavior, with a shape recovery ratio of 86% and shape fixation of 95% as shown in Figure 8F. Practical applications include deformable sealing rings for easy deployment, high modulus springs, and smart molds for complex fiber composition fabrication [50].

The tensile strength of these DLP-printed structures surpasses that of other SMPs tested, including polylactic acid, polyimide, acrylate, and epoxy resin. The SME was confirmed by the *T_trans_* range of the IPN materials (164.8 °C to 175.7 °C). Due to their high modulus, elevated transition temperatures, and robustness under harsh conditions, cyanate-based SMPs are particularly well-suited for applications, such as smart molds and actuators, that require strong temperature responsiveness and high stress output [50].

In the synopsis, these studies reveal a trend in DLP-printed SMPs, suggesting that photothermal constructs favor degradability and remote actuation, but exhibit poor mechanical properties. In contrast, lattice SMPs leverage geometry to achieve functional performance. Lastly, cyanate ester-based SMP systems prioritize strength and thermal endurance over biocompatibility.

### 3.3. Comparative Analysis

Although both SLA and DLP use UV light to cure resin into solid structures, they differ significantly in their curing mechanisms. SLA relies on a focused laser to cure the resin in a continuous and highly precise manner, whereas DLP uses a digital light projector to simultaneously cure entire layers at once. In addition, SLA printing typically employs a wavelength of 405 nm, while DLP printing can utilize both UV and visible light for photopolymerization. A study by Mason et al. (2025) demonstrated that at 365 nm UV light, both cationic (epoxy-based) and radical (acrylate-based) polymerizations can be triggered, while at 405 or 460 nm visible light, only radical polymerizations occur [51]. A comparative analysis of SLA and DLP is given in Table 1.

Both SLA and DLP printing techniques support the production of multi-responsive and tunable structures through variations in resin formulations or the incorporation of fillers. These capabilities enable the design of highly detailed structures on both micro- and macro-scales with rapid speed and high precision. Furthermore, customizable resins allow for mechanical behavior of 3D-printed objects to closely resemble various physiological tissues from rigid bone to soft elastomers, therefore enhancing their suitability for biomedical applications.

However, both printing techniques require post-processing steps, such as post-washing to remove unreacted monomers from the final prints. Solvents like isopropyl alcohol are commonly used for this purpose. While effective in removing excess resin, this step presents a challenge for drug-eluting devices, as the solvent may also wash away embedded therapeutic agents, compromising drug loading and efficacy [52]. SLA-printed objects also require thermal or UV post-curing due to their laser focused streamlined approach, which leaves residual uncured resin. In contrast, DLP cures entire layers uniformly, potentially reducing or eliminating the need for such curing steps [53].

From a biomedical standpoint, DLP has demonstrated remarkable versatility, including applications in gastrointestinal tissue regeneration, the development of SMP dental aligners, and neurological scaffold applications for spinal cord injury repair. Meanwhile, SLA-printing has established a niche in pharmaceutical applications, notably in the fabrication of diagnostic and therapeutic microneedles.

Despite these advances, some flaws remain unaddressed in current research. For instance, the inclusion of nanofillers, such as carbon fillers and multi-walled carbon nanotubes, has expanded the tunability of printing structures by tailoring the degree of crosslinking, thermal stability, and mechanical properties [54]. However, the cytocompatibility of these nanocomposites remains insufficiently explored. Most studies focus on their physical properties, while neglecting toxicity evaluations, limiting their immediate translation into biomedical contexts.

Moreover, the growing interest in sustainability has led to the investigation of closed-loop 3D printing systems through depolymerization of printed structures, as seen in the work by Lopez de Pariza [55]. However, this innovation primarily focuses on entire printed objects and does not directly address the supports used during the printing process, which are critical for structural stability during printing of complex geometries, but are difficult to remove post-printing and may damage the surface of delicate structures [56]. Future work could investigate strategies for selectively depolymerizing supports from bulk polymer while preserving the primary structure, ultimately improving the sustainability and practicality of SLA and DLP technologies.

**Table 1:** Comparison of SLA and DLP printing.

Attribute	SLA (Stereolithography)	DLP (Digital Light Processing)	Biomedical Implications	References
Resolution	High spatial resolution, typically around ~25 mm, suitable for fine feature detail	Slightly lower spatial resolution, around ~50 µm, sufficient for most biomedical applications	SLA better for microneedles, micro-stents; DLP sufficient for scaffolds, dental aligners	[57,58,59]
Printing Speed	Slower due to point-by-point laser scanning curing	Faster, cures entire layers via projected light	DLP preferred for large scaffolds or bulk constructs	[57,60]
Material Compatibility	Primarily acrylate-based resins including polyurethane acrylates with tunable mechanical/thermal properties	Broader resin compatibility including ceramics, metals, elastomers, and hydrogels	Essential SMP performance metrics	[57,58]
Post-Processing	Requires extensive UV or thermal post-curing and solvent washing to remove uncured resin	Often requires minimal or no curing; washing steps to remove residual resin	SLA-printed SMPs need further curing steps to maintain properties	[58]
Application Suitability	Well suited for high precision/small-scale applications, such as microneedles and implant models	Widely used for tissue engineering, dental aligners, flexible biomedical devices	SLA for rigid precision; DLP for flexible, scalable devices	[58]
Printing Volume/Scale	Typically smaller build volumes due to slower scanning	Larger volume printing enabling manufacturing of bigger constructs	DLP more scalable for biomedical scaffolds	[60]
Shape Fixity Ratio (*R_f_*)	SLA-printed tBA-co-DEGDA polymer shows high shape fixity, though exact values are not disclosed	Electrical stimulus- activated DLP SMP composites demonstrate *R_f_* ≈ 100%	SLA SMPs reliably hold temporary shapes which are critical for deployment; DLP composites enable nearly perfect fixation, ideal for precise biomedical actuation	[61]
Shape Recovery Ratio (*R_r_*)	SLA polymers show high shape recovery performance over multiple cycles (implicitly >95%), but exact *R_r_* values are not specified	DLP CNT/SMPCs demonstrate *R_r_* > 95%	Both methods offer reliable recovery; DLP composites especially promising for highly accurate biomedical actuation	[61]
Surface finish	Usually smoother finish due to laser focus	Slightly rougher; limited by pixel size of projected light	SLA best for smooth surgical implants	[62]
Cost and Accessibility	Generally higher cost and complexity	More affordable and simplified optics	DLP is more accessible for biomedical labs	[60]

Table 2 provides an overview of shape memory resins utilized in SLA and DLP printing, including quantification of SME, mechanical properties, and biocompatibility.

## 4. Recent Advances in SMP Chemistry for DLP and SLA

### 4.1. Novel Photoinitiators (BAPO, TPO-L) for Cytocompatibility

Diphenyl(2,4,6-trimethylbenzoyl)phosphine oxide (TPO) is a type I acylphosphine oxide-based photoinitiator (PI) widely used in DLP 3D-printing due to its ability to initiate polymerization without the need for co-initiators, making it a standalone PI system [83]. TPO absorbs light in the wavelength range of 368–425 nm and appears as a light-yellow powder capable of generating free radicals under UV exposure, triggering rapid crosslinking between monomers in resin formulations [84]. This lack of control of crosslinking speed, however, was addressed by Zhang et al. (2014), who successfully pre-dissolved TPO in poly(ethylene glycol) diacrylate (PEGDA) to fabricate photocurable, 3D-printable hydrogels [85].

In comparison to other common PIs, TPO also demonstrates favorable cytocompatibility. A study using L-929 mouse fibroblasts revealed that TPO yielded a cell viability of 84.45 ± 3.62%, outperforming phenylbis (2,4,6-trimethylbenzoyl) phosphine oxide (BAPO), which showed lower viability at 74.16 ± 3.7%, though slightly underperforming ethyl (2,4,6-trimethylbenzoyl) phenylphosphinate (TPO-L), which exhibited 89.62 ± 4.93% [86].

Advances in PI design led Xie et al. [87] to develop modified acylphosphine oxide-based self-floating PIs such as TPO-F, TPO-Si, C12, and C22. These PIs were developed to mitigate oxygen inhibition at the resin surface. Among these, TPO-Si and TPO-F demonstrated superior migration behavior attributed to their low surface energy, allowing for fast surface curing by preventing oxygen diffusion deeper into the resin. The versatility of TPO was further demonstrated by Aldana et al. (2024), who incorporated 1 wt% of this PI into a poly(caprolactone-co-trimethylenecarbonate) urethane acrylate resin to develop SMPs for DLP, creating functional, actuating structures, such as claw-like constructs [67].

**Figure 9:**
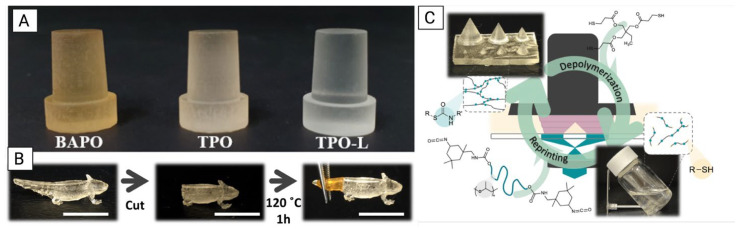
(**A**) 3D-printed specimens with different photoinitiators. Reprinted from Ref. [86]. (**B**) Repair of axolotl’s tail by the addition of self-healing. The scale bar represents 200 micrometers. Reprinted from Ref. [55]. (**C**) Reaction scheme of the depolymerization and DLP printing of PSU materials. Reprinted from Ref. [55].

**Figure 10:**
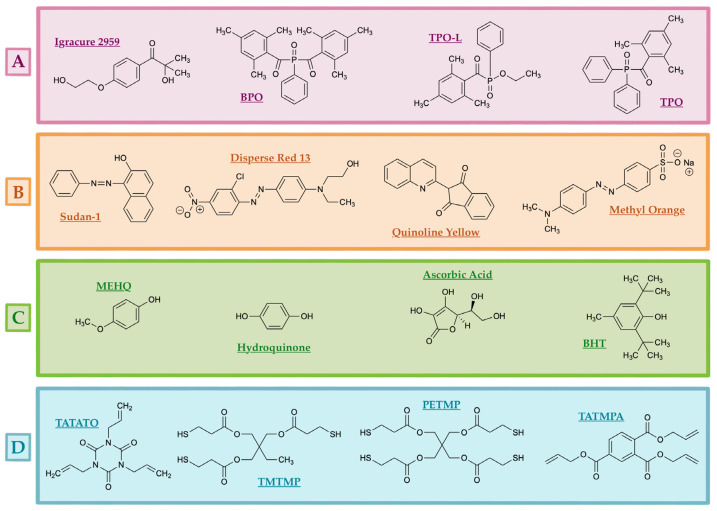
(**A**) Photoinitiators: 2-Hydroxy-4′-(2-hydroxyethoxy)-2-methylpropiophenone (IGRACURE 2959, PubChem CID: 86266, CAS No. 106797-53-9), Bis(2,4,6-trimethylbenzoyl)phenylphosphine oxide (BPO, PubChem CID: 164512, CAS No. 162881-26-7), Ethyl (2,4,6-trimethylbenzoyl)phenylphosphinate (TPO-L, PubChem CID: 16205480, CAS No. 84434-11-7), (2,4,6-Trimethylbenzoyl)diphenylphosphine oxide (TPO, PubChem CID: 166480, CAS No. 75980-60-8) (**B**) Photoabsorbers: 1-(Phenylazo)-2-naphthol (Sudan-1, PubChem CID: 13297, CAS No. 842-07-9), 2-[4-[(2-chloro-4-nitrophenyl)diazenyl]-*N*-ethylanilino]ethanol (DISPERSE RED 13, PubChem CID: 18516, CAS No. 3180-81-2), 2-quinolin-2-ylindene-1,3-dione (QUINOLINE YELLOW, PubChem CID: 6731, CAS No. 8003-22-3), Sodium 4-[4-(Dimethylamino)phenylazo]benzenesulfonate (METHYL ORANGE, PubChem CID: 87058815, CAS No. 547-58-0) (**C**) Photoinhibitors: 4-Methoxyphenol (MEHQ, PubChem CID: 9015, CAS No. 150-76-5), 1,4-Dihydroxybenzene (HYDROQUINONE, PubChem CID: 785, CAS No. 123-31-9), L-ascorbic acid (ASCORBIC ACID, PubChem CID: 54670067, CAS No. 50-81-7), 2,6-Ditert-butyl-4-methylphenol (BHT, PubChem CID: 31404, CAS No. 128-37-0) (**D**) Monomers: 1,3,5-tris(prop-2-enyl)-1,3,5-triazinane-2,4,6-trione (TATATO, PubChem CID: 13931, CAS No. 1025-15-6), Trimethylolpropane tris(3-mercaptopropionate) (TMTMP, PubChem CID: 118379, CAS No. 33007-83-9), Pentaerythritol tetrakis(3-mercaptopropionate) (PETMP, PubChem CID: 82056, CAS No. 7575-23-7), Triallyl trimellitate (TATMPA, PubChem CID: 75903, CAS No. 2694-54-4).

In contrast, BAPO, another type I PI, also suffers from poor solubility in various monomers and oligomers but has the unique advantage of generating up to four radicals per molecule, double that of TPO, which in turn leads to enhanced light absorption and polymerization efficiency. However, BAPO tends to cause undesirable discoloration in resin systems as shown in Figure 9A, and exhibits higher cytotoxicity than TPO at equivalent concentrations [88]. It operates within a slightly narrower absorption range of 365–416 nm [89]. Resin component chemical structures can be visualized in Figure 10.

### 4.2. Low-Shrinkage, High-Recovery Thiol-Ene Resins

Recent developments in low-shrinkage, high-recovery thiol-ene resins have demonstrated promising potential for repairable, sustainable, and shape-morphing 3D-printed structures. Lopez de Pariza et al. [55] introduced dynamic covalent bonds (DCBs) into a poly(thio)urethane (PSU) thermoset resin by leveraging the click-reaction between isocyanates (R’-NCO) and thiols (R-SH), forming thiourethane (R-S-CO-NH-R’) linkages within the polymer backbone. This approach enabled a closed-loop photoprinting system, where printed PSU structures could be chemically depolymerized into thiol-terminated monomers for future reprinting. The depolymerization cycle is shown in Figure 9C. Additionally, the PSU resin demonstrated repairability through thermally activated DCBs, in which damage to printed structures could be healed via heating, as demonstrated in Figure 9B where a printed axolotl model had its tail removed, reprinted, and successfully reattached after heating at 120 °C for one hour [55].

In another investigation, Shaukat et al. [64] developed a database of thiol-acrylate resins for DLP vitrimers by combining mono- and bi-functional acrylates with thiol crosslinkers of varying ester content and functionality. Incorporating tri- and tetra- functional acrylates allowed for the creation of highly crosslinked vitrimer networks, and mechanical and thermal properties were systematically studied. Notably, when 2-hydroxy-3-phenoxy propyl acrylate (HP1A) ethylene glycol bis-mercaptoacetate (EGMA) and ethylene glycol bis(3-mercaptopropionate) (EGMP) displayed superior tensile strength compared to the resin formulation involving the thiol 1,6 hexane dithiol (HXDT). More precisely, the improvement in toughness for the resin formulations EGMA-HP1A and EGMP-HP1A (15 MPa) compared to HXDT-HP1A (9 MPa), was attributed to the reduction in shrinkage stress and thermo-induced rearrangements of the network [64].

Expanding on this work, Shaukat et al. [90] synthesized thermally responsive thiol-acrylate vitrimers using acrylated epoxidized linseed oil (AELO) and evaluated the effect of thiol functionality. The trifunctional thiol trimethylolpropane tri(3-mercaptopropionate) (TMTMP) resulted in a more highly crosslinked polymer (*T_g_* = −18 °C), while AELO-EGMP networks exhibited greater bond exchange kinetics at elevated temperatures (>140 °C) due to their lower crosslinking density. Demonstrating the practical potential of these materials, the authors fabricated a 3D-printed flower exhibiting triple shape memory behavior. By leveraging the *T_g_* and the topological freezing temperature, two different shapes were programmed, and the first temporary shape recorded a recovery time of 30 s under thermal stimulus [90].

### 4.3. Multi-Responsive SMP Systems (Thermo + pH, Thermo + NIR Light)

Multi-responsive SMP systems that respond to various stimuli such as temperature, pH, and NIR light have shown remarkable potential for programmable, self-healing, and highly reversible actuation. Wang et al. [91] developed thermal and NIR-triggered triple shape memory polymers by blending poly(lactic) acid (PLA) and polycaprolactone (PCL), then embedding polypyrrole (PPy) nanoparticles to produce a PLA/PCL/PPy nanocomposite. These composites were able to adopt two temporary shapes in addition to their manufactured, permanent conformation, giving rise to triple SMP behavior.

The optimal shape memory performance was achieved with an 80:20 PLA/PCL ratio, yielding recovery rates between 85–93%. The addition of PPy, a photothermal conversion filler, enhanced the nanocomposite’s NIR responsiveness, resulting in rapid shape recovery ratios exceeding 95%. Under NIR exposure, localized melting and recrystallization within the polymer phases enabled reversible transitions between programmed shapes. Moreover, the nanocomposites exhibited excellent self-healing efficiencies above 90%, demonstrated by the complete restoration of fractured samples through NIR-induced chain reconnection [91].

In another study, Peng et al. [92] created a dual-responsive hydrogel via a cellulose-fiber-based napkin with UV-curable adhesion. This bi-layer system could be programmed in warm water, representing the physiological temperature of the body (37 °C), fixed in cold water (5 °C), and further actuated by pH or thermal triggers. Fixity tests displayed *R_f_* around 87–88% depending on the configuration in which the SMP is bent. The actuator exhibited pH sensitivity, returning to its original shape at pH = 3 and displaying increasing bending angles as pH values rose. When combined with thermal stimulus, the system demonstrated a two-way shape memory effect (SME) in warm water, followed by tuning of the pH [92].

Similarly, Fan et al. [93] fabricated dual-responsive SMP composites using azobenzene derivatives (ADs) as molecular switches loaded in a polystyrene-block-poly (ethylene-co-butylene)-block-polystyrene (SEBS) elastomer matrix. While SEBS naturally exhibits thermally induced shape memory due to transient crosslinking from its poly (ethylene-co-butylene) (PEB) segments, the incorporation of ADs introduced photoresponsiveness via UV-induced isomerization. Adjusting alkane side chains in the ADs allowed for customization of melting temperatures, thus enhancing thermal responsiveness. The resulting composites preserved the high stretchability of pristine SEBS, with fracture strains exceeding 900%, and enabled 2D and 3D rapid shape recovery within 2 s of UV exposure [93].

### 4.4. Inclusion of Nanofillers (Graphene, Cellulose Nanocrystals)

The loading of nanofillers into SMPs has emerged as a compelling strategy for tailoring thermal, mechanical, electrical, and shape memory properties in 3D-printable objects. Badria et al. [94] optimized thiol-ene thermosets derived from thiol monomers and rigid trizaine-trione (TATO) for SLA printing by enhancing their formulation with additives of either a free radical inhibitor (pyrogallol), or the addition of a photo-absorber (Sudan I) and diluent (PETMP). The ideal formulation (thermoset 1), combining TATO monomers, tris[2-(3-mercaptopropionyloxy)ethyl]-isocyanurate (TATO thiol) and 1,3,5-tiallyl-1,3,5-triazine-2,4,6-trione (TATO alkene), demonstrated reduced curing depth and improved thermal stability. PETMP at 17 wt% was the minimum concentration to achieve SLA printing, and effectively reduced viscosity, thus facilitating smoother printing. Mechanical property tests showed that printed thermosets containing pyrogallol or Sudan I achieved stiffness values of 1.85 GPa and 1.6 GPa, respectively, ultimately surpassing all previously published acrylate-free thiol-ene SLA resins. Furthermore, the inclusion of nano- and micro-scale hydroxyapatite (HA) particles elevated the modulus, suggesting promising potential for printing bone fixation implants. A trade-off associated with obtaining a higher modulus is a reduction in printing resolution caused by the incorporation of HA particles. Surprisingly, there was no drastic difference in cytocompatibility among formulations comprised of either additives or HA particles, as all formulations were non-toxic [94].

In another study, Mu et al. [95] employed DLP to fabricate electrically conductive SMP composites by embedding multi-walled carbon nanotubes (MWCNTs) into an acrylic-based photocurable resin. Furthermore, the optimal conductivity was achieved at 0.3 wt% MWCNT loading, enabling sufficient and quick electro-induced recovery within 2 s under 180 V. This formulation enabled the printing of stretchable circuits, electro-responsive SMPs, and capacitive sensors. While MWCNTs improved the modulus and ultimate tensile stress by 45% and 21%, respectively, they slightly reduced elongation at break by 10%, confirming a beneficial trade-off between mechanical strength and ductility. Increasing filler content beyond this threshold would improve conductivity at the expense of strain tolerance and flexibility, limiting feasibility in high deformity applications [95].

**Figure 11:**
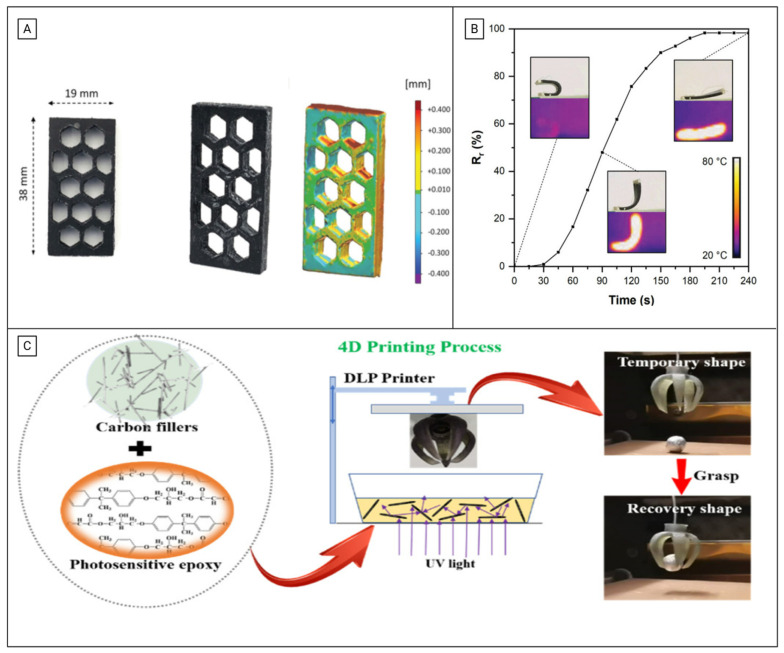
(**A**) 3D-printed honeycomb structure and corresponding 3D scanning deviation heat map. Reprinted from Ref. [96]. (**B**) Snapshot of Joule heating-induced shape recovery recorded by a thermal camera. Reprinted from Ref. [96]. (**C**) DLP printing of photosensitive composite material, comprehensive fabrication, and application of photosensitive composite ink. Reproduced with permission from [54].

Similarly, Cortés et al. [96] developed electro-responsive SMPs using MWCNTs embedded in a poly(ethylene glycol) diacrylate/poly(hydroxyethyl methacrylate) (PEGDA-co-PHEMA) matrix, confirming that Joule heating from electrical stimulation could remotely activate the SME. High fixity (*R_f_* > 100%) and recovery ratios (*R_r_* > 95%) were achieved, with temperature elevation directly proportional to MWCNT content. Figure 11B presents joule heating as a trigger for shape recovery. In addition, voltages of 160 V and 250 V were required to reach the *T_g_* for 0.5 wt% and 0.3 wt% MWCNT composites, respectively. This highlights a recurring contradiction in electro-activated SMPs: lower filler concentrations preserve matrix conformability and potential cytocompatibility but require higher activation voltages, whereas higher loading minimizes electrical necessity at the sacrifice of reduced elongation and increased stiffness [96].

Additionally, Wang et al. [54] formulated UV-curable inks composed of PEGDMA, epoxy acrylate (EPAc), and carbon fillers. Introducing EPAc into the PEGDMA-based system across exposure time significantly increased the crosslinking degree, improving tensile strength by 10.6 MPa while slightly reducing strain. However, higher crosslinking densities and filler embedding accelerated thermal degradation due to C-O bond scission, indicating lower thermal stability and revealing a caveat between mechanical reinforcement and long-term thermal security. Moreover, increasing UV exposure times further boosted crosslinking density and bolstered the tunability of these materials.

Collectively, these studies showcase that the strategic inclusion of nanofillers enables fine-tuning of SMP mechanical properties. On the other hand, these fillers also introduce conflicting constraints associated with ductility, voltage activation, and thermal stability. Therefore, identification of optimal filler concentrations and exposure times remains a critical challenge in applications involving deformable matrices utilizing electrical stimulus. Figure 11C depicts the comprehensive process of the photosensitive composite inks, beginning with its composition, fabrication, and finally application as a claw-like construct [54].

## 5. Biomedical Applications of 3D Printed SMPs

SMPs are a unique class of materials explored for their use in biomedical applications, due to their highly tunable properties, such as biodegradability, shape fixity, and responsiveness to external stimuli. Since these materials come into contact with body tissues and fluids, these materials must not exhibit immunogenicity. To be employed safely in biomedical contexts, SMPs must meet several requirements relating to biocompatibility and non-toxicity.

### 5.1. Dental Technology

One widely known and implemented application of DLP printing is in the dental industry, where DLP printers are used to fabricate the widely known Invisalign technology for orthodontics. As clear aligners became increasingly popular among practitioners and patients, the use of DLP technology has become an efficient method to precisely print patient-specific constructs, utilizing intraoral scanners and computer-aided design (CAD) design software [97]. DLP printing allows orthodontists to manage treatments in-office, allowing for improved efficiency and sustainability.

However, resin formulation must be optimized for the oral environment, and not all DLP resins are biocompatible. In a study by Atta et al., material analysis of both shape memory aligners and traditional aligners was conducted, including thermoformed CA Pro (CP) (polyethylene terephthalate (PET) or polyethylene terephthalate glycol (PETG) composition), Zendura A (ZA) (polyurethane resin), Zendura FLX (ZF) (polyurethane (TPU) composition) and SMPs that were 3D-printed Graphy Tera Harz TC-85 (TC-85) (polyurethane and acrylate compounds). Out of these materials, the SMP showed the best adaptability by applying the most consistent force to the teeth over the study duration, which is crucial for alignment. Although Tera Harz TC-85 appears to be a promising SMP for 3D printing in orthodontic applications, further research is needed to optimize resin formulation and ensure long-term safety for the patient [98].

Strunz et al. noted that DLP printers can be used to produce dental implant models. Traditionally, models of the patient’s teeth would be made by taking an impression with silicone, filling with plaster, and fixing any defects present in the final cast by hand. This method may lack precision and relies heavily on the specialized training and experience of the technician. Comparatively, this study demonstrates DLP printing using resin formulations can achieve high precision implant models with less waste and precise customization using patient intraoral scans, with a lower standard deviation in print accuracy than traditional methods and even SLA printing. Despite these benefits, this process must be optimized for use in a clinical setting, including standardization of printers and calibration settings [99].

Another dental application using DLP printers includes the fabrication of novel multi-layered, multi-material dentures, crowns, and bridges. In a study by Schweiger et al., researchers utilized 4D-printing techniques to fabricate dental implants from scans of extracted teeth, starting with the dentine core Objet VeroGlaze MED620 (Stratasys Ltd., Rehovot, Israel). and layering the incisal area on top Objet MED610 (Stratasys Ltd., Rehovot, Israel) using biocompatible acrylate polymers. The resulting teeth demonstrated exceptional light dynamic results, but showed a limitation in surface qualities due to printing orientation and scaffolding [100]. However, through further development, the findings suggest that a bilaminar technique could be a viable approach to achieve fast, reproducible, and aesthetically pleasing dental restorations.

### 5.2. Drug Delivery Applications

DLP can allow for the precise fabrication of pharmaceutical devices, specifically microneedles and site-specific drug delivery systems. To illustrate this, a study conducted by Loh et al. utilized DLP and SLA to fabricate microneedles, an emerging technology used in various procedures, therapeutics, disease monitoring, and diagnostics. Traditional methods of micro-molding are unable to achieve customization, and thus, vat polymerization can be used to enable high specificity and improved performance of microneedles. DLP, SLA, and continuous liquid interface production (CLIP, first used in 2015 to grow polymers from a resin pool rather than printing layer-by-layer to provide the means for precise customization) are used to achieve intricate geometries, dimensions, and architectures. Thus, fabrication with higher dimensional accuracy can accommodate patient skin topography, integrating advanced features like microchannels, which can enhance drug permeation and allow for more effective management of chronic conditions [101].

In another study conducted by Kadry et al., researchers assessed the use of DLP technology to fabricate modified-release tablets using photoreactive polymers, which enable precise control over rate, time, and location of drug release in the body, ultimately providing more consistent long-term treatment for various diseases and disorders. By leveraging DLP printing technology, researchers were able to fine-tune parameters, including tablet dimensions, UV light intensity, and exposure duration, to fabricate an oral dosage form of theophylline. In addition to the initial model drug, DLP technology can be extended to a broad range of thermostable and thermolabile drugs. However, some limitations include poor solubility of some drugs within photopolymers, the potential for drug degradation during UV curing, and regulatory concerns regarding the safety of UV-curable polymers in pharmaceutical applications. Building upon this study, future directions aim to develop safe photoreactive polymers for accommodating both hydrophilic and hydrophobic drugs, and thus, expanding the use of DLP technology in pharmaceutical manufacturing [102].

**Figure 12:**
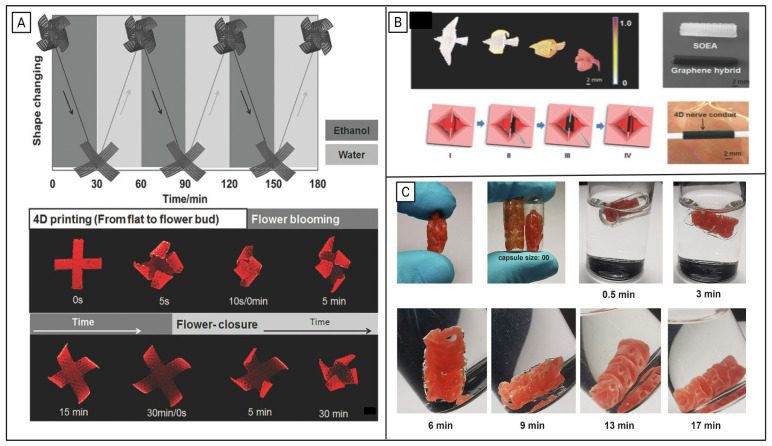
(**A**) Dynamic process of 4D-printed flower with reversible shape change. Reproduced with permission from [104]. (**B**) This figure demonstrates a graphene-enhanced 4D-printable nanohybrid material that enables programmable curvature for fabricating reprogrammable nerve guidance conduits (NGCs) to aid severed nerve regeneration. Reproduced with permission from [104]. (**C**) Programmed 3D-printed enteric capsule in comparison to its original shape, followed by incubation in water at 37 °C. Reprinted from Ref. [103].

Similarly, Yuts et al. engineered 4D DLP-printed expandable enteric capsules based on poly(β-aminoesters) that can improve the bioavailability of macromolecular drugs across the GI tract (Figure 12C). As this system can be compressed into digestible, biodegradable capsules, it has the potential to lower intestinal blockage and prevent mechanical obstruction in patients with high risk of GI stenosis. The capsules demonstrate good cytocompatibility, allowing for the material to be a promising material for biomedical applications beyond the gastroenteric system (Figure 12A) [103].

**Figure 13:**
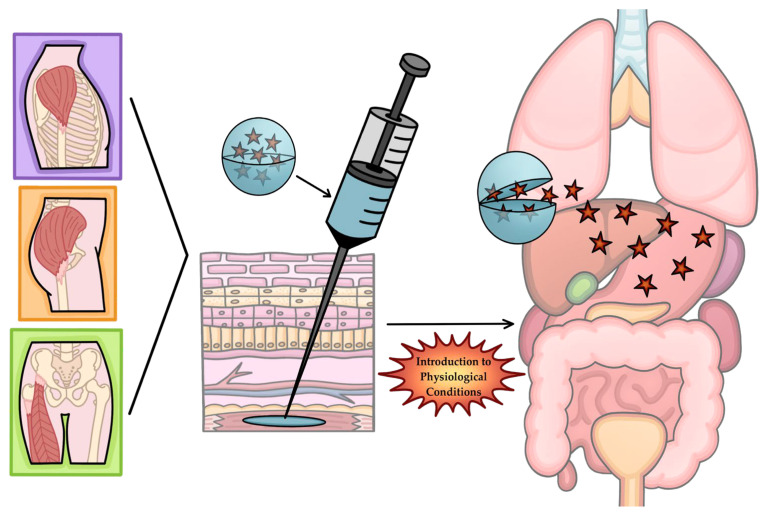
Site-directed delivery of drugs within SMP, triggered by introduction to physiological conditions, such as pH, temperature, and/or hydration.

Another way SMPs can be utilized in drug delivery is supported by Adamov et al., where researchers were able to print immediate-release tablets using DLP technology for incorporating highly potent drugs, like zolpidem tartrate, into more flexible dosages [105]. The tablets were printed in PEG 400, and further characterized to evaluate drug release from the polymer matrix. Conclusions from the study demonstrated that the accessibility and customizability of DLP technology and the optimization of polymer formulations allow for personalized dissolution profiles to aid in the treatment of chronic illness. The general mechanism for site-directed drug delivery using SMPs is illustrated in Figure 13.

### 5.3. Nervous System Applications

Due to the complexity of the nervous system, the efficiency and precision of DLP is beneficial for tissue engineering and surgical planning. Specifically, spinal cord injuries have proven challenging to repair due to poor nerve regeneration in vivo. Thus, DLP printing can be utilized to fabricate intricate structures that facilitate tissue regeneration. Yuan et al. summarized the requirements for a successful scaffold, establishing the following criteria: accurate conformity to the spinal cord’s dimensions mechanical properties, facilitation of cell adhesion, proliferation, and differentiation, biocompatibility, and predictable biodegradability [106]. For example, in a study by Kwokdinata et al., researchers were able to successfully fabricate GelMA and GelMA-PEGDA-based tissue scaffolds using DLP printing to aid in the regeneration of spinal cord progenitor cells, improving the prognosis of spinal cord injury. Utilization of DLP printing technology allows for scaffolds with high microchannel density and the low mechanical stress, increasing cell viability and allowing embedded cells to retain functional characteristics essential for tissue engineering applications. However, increasing PEGDA concentrations showed potential cytotoxicity, and this limitation must be addressed before its application in a clinical setting [107]. Furthermore, future studies are needed to optimize construct architecture and degradation rates.

SLA printing can also be used to fabricate nerve conduits from responsive SMPs. This is detailed in a study by Miao et al., where researchers explored the thermomechanical programmability of a naturally derived soybean oil epoxidized acrylate (SOEA) for use in 4D-bioprinting [108].

### 5.4. Orthopedic Applications

DLP printing can also be used in orthopedics, with applications in implant integration and bone grafts. Zhang et al. addressed distal finger regeneration using multi-scale, porous 3D DLP-printed bioceramics fabricated using calcium phosphate (CaP). First, they used SolidWorks (Version 31) to design internal structure features based on patient CT scans, then printed the scaffolds with a PEGDA-400 photosensitive resin. Cells were shown to thrive on the construct surface, exhibiting osteogenesis in vitro. Furthermore, 3D-printing technology enabled precise control over physicochemical properties, including surface roughness, internal grain size, micropore interconnectivity, protein adsorption, and ion release [109]. Thus, the optimization of these scaffolds shows promise in clinical applications for isolated bone tissue regeneration.

Similarly, in another study by Wang et al., a liquid poly(4-methyl-ε-caprolactone) dialkynylate (PMCLDY) polymer was prepared as a DLP-printable ink for customized tissue scaffolding (Figure 12B), demonstrating elasticity and mechanical strength. The scaffolds were formulated to be biodegradable, supporting the growth and proliferation of bone marrow-derived mesenchymal stem cell (BMSCs) for osteogenesis.

### 5.5. Cardiovascular Applications

SLA printing can be used to fabricate vascular models for medical training and pre-procedural planning. Specifically, these models are useful in endovascular practice, offering haptic feedback within a highly accurate anatomical simulation that allows physicians to practice with guide wires, catheters, and stent grafts. Through the use of thiol-ene based resins, the model achieves mechanical properties similar to human aortic tissue. However, soft resins are prone to printing failures, and require post-processing steps, including washing and post-curing to remove residual resin, that may increase the mechanical strength of the constructs and reduce the biomimetic accuracy of the model.

In a similar study done by Henriques et al., this concept was built upon through the development of patient-specific models for the procedural planning of percutaneous angioplasty. These models, like the ones above, were SLA-printed, but were composed of materials that more accurately replicated cardiovascular tissue [110].

A review from Hua et al. illustrated that DLP and SLA technology can be employed to fabricate cardiovascular stents. For example, stents made from PLA and PCL can be used to widen blocked blood vessels, while gradually degrading as the vessel heals and the blockage resolves. DLP and SLA printing allow for efficient manufacturing of various sizes and designs, however, they may still face challenges with low printing resolution [111]. Furthermore, the biodegradability of the polymer construct can help alleviate long-term side effects due foreign body response. However, further studies are needed to optimize radial stiffness and axial flexibility during vascular remodeling.

### 5.6. Other Applications

**Figure 14:**
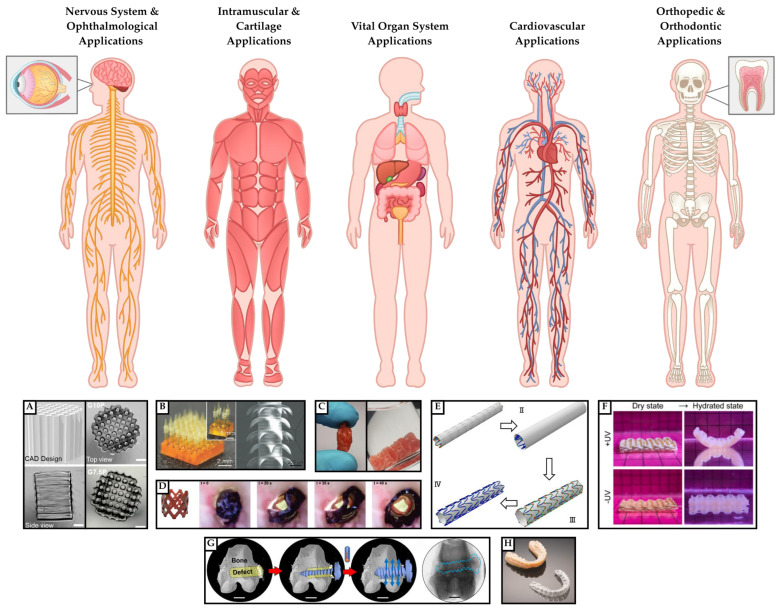
Applications of SMPs in biomedical engineering. (**A**) Bioprinted microchannel scaffolds to modulate neuronal differentiation for spinal cord injury repair (scale bar 500 µm). Reproduced with permission from [107]. (**B**) Bio-inspired microneedles with 4D-printed deployable barbs to enhance tissue adhesion. Reprinted from Ref. [101]. (**C**) 4D DLP-printed expandable enteric capsules. Reprinted from Ref. [103]. (**D**) NIR-mediated expansion of 4D-printed stent for thermal destruction of malignant cells in esophagus or intestines. Reprinted from Ref. [47]. (**E**) Biodegradable, compressible 4D-printed cardiac stent. Reprinted from Ref. [111]. (**F**) Deployment of flat-to-curved 4D-printed SMP architecture for minimally invasive bone graft. Reprinted from Ref. [109]. (**G**) Repair of bone defect with 4D-printed polymeric screw (scale bar 5 mm). Reprinted from Ref. [109]. (**H**) SMP dental aligner fabricated via additive manufacturing. Reprinted from Ref. [97].

In a study published by Paunovic et al., researchers used a DLP-printed photopolymerizable composite to fabricate a stent with NIR light-triggerable shape transformation to treat tumors in nonvascular organs, like the esophagus. The developed stent, composed of polyester copolymer with embedded gold nanorods, demonstrated high printing resolution, efficient light-triggered shape recovery, and degradability under physiological conditions [47]. However, despite their potential, the proposed stents need to be further optimized to improve mechanical resilience to mimic anatomical structures. The applications of DLP and SLA-printed SMPs are summarized below in Figure 14 and Table 3.

## 6. AI in SMP Development

**Figure 15:**
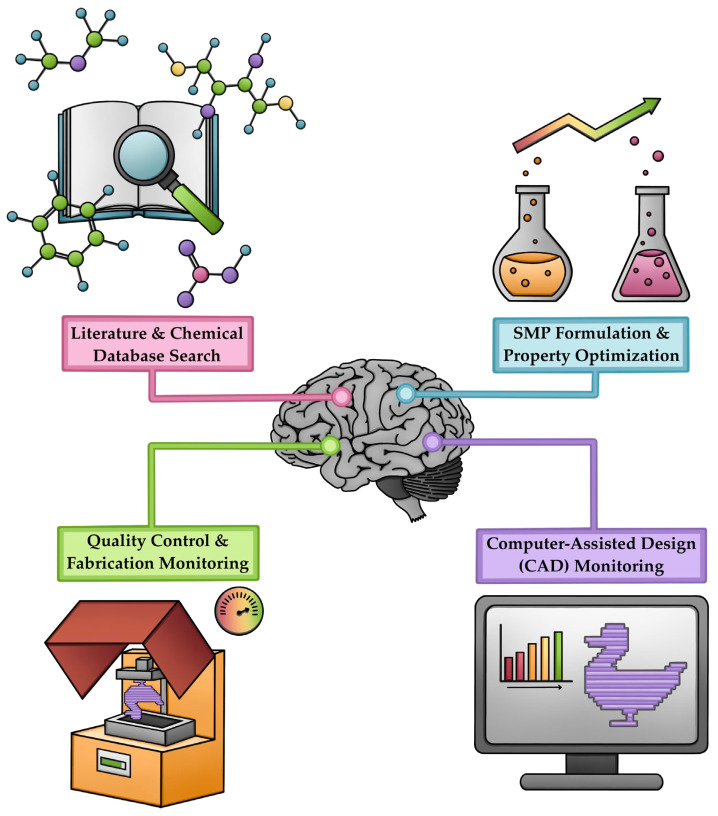
AI integration in SMP formulation and fabrication.

Artificial intelligence (AI) plays a pivotal role in the development of shape memory polymers through advanced fabrication techniques, addressing challenges in SMP formulation, biocompatibility, and evaluation of long-term material properties. AI enhances the optimization of polymer performance, accelerating the development process, and ensuring quality is maintained for use in clinical applications (Figure 15).

### 6.1. AI-Driven SMP Design and Printing via SLA/DLP for Biomedical Applications

The integration of AI with SLA and DLP printing platforms will revolutionize the design and production of SMPs, specifically for biomedical applications. Together, they are yielding new avenues for material design, process efficiency, and quality assurance, and, thus, establishing a new pathway for the advancement of 4D-printing as it pertains to healthcare. The complexities of SMP behavior and the process parameters required for vat photopolymerization techniques create an opportunity for intelligent computation to help us navigate future development [117,118,119].

### 6.2. AI-Driven Material Discovery and Design

In one study, by Yan et. al [120] built various systems that incorporated BigSMILES notation, mixed-dimensional input architectures, and dual-convolutional neural networks for the prediction of recovery stress and glass transition temperature of thermoset SMPs, which successfully screened 14 new formulations from large chemical libraries. The identified AI and machine learning methodologies successfully utilized transfer learning, variational autoencoders, and weighted vector combination procedures to design ultraviolet-curable SMPs with the desired properties [120].

Physics-informed neural networks (PINNs) offer a useful approach to estimate material properties, as they incorporate a Priori physical laws and model-data integration, resulting in enhanced performance with generally fewer initial experimental measurements for optimization. PINNs allow for the prediction of SMP behavior within different physiological environments, which is useful in the biomedical space [117,118,121].

Neural network models can incorporate structural relaxation properties, while considering the dynamics of phase transitions to predict shape recovery behavior, fixity ratios, and mechanical response under relevant programming conditions. These models will continue to improve with further data collection, and formulations can then be optimized to ensure maximum shape memory performance at body temperature (37 °C). [67,121,122,123,124].

Machine learning has also enabled more accurate and efficient characterization of SMP behavior using video data analysis, with reported 90% sensitivity and 94% specificity in measuring material behavior. This approach greatly reduces the time for material development by giving immediate feedback of shape recovery characteristics during experimental testing [125,126].

### 6.3. Process Optimization and Parameter Control

The integration of artificial intelligence in SLA and DLP processes has addressed important challenges in manufacturing precision and consistency. Machine learning algorithms can characterize photopolymerization parameters (exposure time, layer thickness, light intensity, and automatic generation of support structures, etc.) with the aim of producing an end-product with high dimensional accuracy and surface quality. Conventional trial-and-error methods are inefficient in the analysis of multiple, interconnected parameters needed to optimize high-quality SMP fabrication [117,127,128,129].

Hierarchical machine learning systems can autonomously adjust printing parameters based on geometric characteristics and mechanical needs, significantly reducing the need for post-processing steps and improving manufacturing efficiency (Figure 15) [127].

Recent studies have been able to train several reinforcement learning (RL) prototyping systems, where fabrication parameters were adjusted in real time based on feedback. More specifically, the adaptive Linear-Quadratic Regulator (LQR) method using Q-learning (ALQ) has been developed for the shape control of 4D-printed SMPs to optimize the morphing behavior based on dynamic stimuli. The flexibility provided by these platforms is beneficial for biomedical applications, where consistent quality is crucial to safety and efficacy [129,130].

### 6.4. Real-Time Monitoring and Quality Control

The advancement of machine vision using convolutional neural network (CNN) architectures enables real-time automated detection of defects (e.g., delamination, porosity, warp, filament issues, etc.) for SLA, DLP, FDM, and metal additive manufacturing. In powder-bed fusion processes, CNNs can classify splatter and delamination with a maximum accuracy of 96.8%, while complications such as cracks and lack of fusion were classified at an accuracy of 92.1%. In fused filament fabrication (FFF) systems, there was a 100% success rate in failure detection for full 3D-model reconstructions. Critical preprocessing techniques that improve detection accuracy include denoising, contrast enhancement, and edge detection, which prove especially crucial for biomedical applications where minor structural defects can compromise the safety of devices [131,132].

The most recent paradigm of AI-augmented additive manufacturing is identified as AI2AM, which provides real-time monitoring, optimizes additive manufacturing parameters to minimize defects, maximizes efficiency, and improves sustainability [133]. In melt electrowriting (MEW) printing, integration of computer-vision, neural networks, and optimization-feedback into a closed-control system has shown target outputs with output error within 5% of the requested value, while continuously adjusting parameters throughout the printing process [134]. A twin framework was employed for deep neural operators and in situ sensor inputs, thereby calculating melt pool states to minimize defects and quantify uncertainties present in Laser Powder Bed Fusion (L-PBF) printing. Thereafter, regularized 4D neural fields based on volumetric approximation successfully performed smooth interpolation of observed geometries for dynamic, data-driven optimization of printed artifacts, while minimizing material consumption and post-processing steps [135].

In the area of 4D printing, closed-loop AI systems rely on sensory feedback with adaptive control of toolpaths. In one study, a hydrogel-based sensor exhibited printing capabilities on a breathing porcine lung, with a toolpath tracking error of only 0.65 mm. This introduces an exciting prospect for furthering the use of robotics in medical manufacturing, potentially even autonomously for surgical applications [118].

### 6.5. Multimodal AI Integration

Advanced AI systems combine many sensing modalities to create a full process monitoring and control solution. Thermal imaging, visible light imaging, acoustic sensing, and force measurements all constitute a total monitoring environment for characterization of complex printing processes. Machine learning algorithms analyze multi-modal data to identify patterns and associations that are otherwise invisible to the streams from each individual sensor [136,137].

The combination of AI with CAD facilitates an automatic generation of the optimized support structure and print orientation, minimizing material waste during manufacturing, while maximizing the surface quality of the printed object [127,128].

Through gradient-descent and evolutionary algorithms utilizing machine learning, efficient material distribution can be employed to achieve desired changes in 3D shape. Such methods can analyze massive design databases extremely quickly, which would otherwise require unrealistic computational resources with finite elemental methods, allowing designers to explore new geometries [119,137].

### 6.6. Current Limitations and Research Needs

Several barriers persist in the full deployment of AI to develop and print SMPs. The lack of available data still hinders model performance, especially for new material formulations and biomedical applications. Since high-quality datasets must be large to train machine learning algorithms, progress will require collaborative research efforts [119].

For AI technologies to be widely adopted, transfer learning strategies that can successfully adjust to new materials and procedures while preserving performance must be developed. Technical issues with processing demand and system dependability arise when integrating AI systems into current manufacturing processes, and research is still being done to create reliable architectures that translate well into industrial settings [137].

AI’s convergence with SLA and DLP technology has transformed the design and manufacturing of SMPs for biomedical applications, and this convergence will enable efficient clinical transfer of 4D-printing into personalized medicine, minimally invasive surgery, and adaptive biomedical devices. As the field advances, more complex AI architectures and richer data sets will improve the precision, quality assurance, and availability of SMP manufacturing.

## 7. Emergence of 4D-Printed SMPs for Biomedical Applications

The 4D-printing of SMPs offers medical practitioners real-time responsiveness to applied stimuli, such as heat, light, moisture, and/or pH. Like 3D-printed constructs, SMP-based 4D-printed materials can change geometry, stiffness, or porosity over time in vivo to create new avenues for tissue repair and drug delivery [138,139].

### 7.1. Self-Fitting Implants and Scaffolds

SMP-based 4D-printed constructs can be deployed compactly via minimally-invasive procedures and subsequently expanded in physiological conditions to take on the geometry of irregular defects. Bone and cartilage scaffolds have been manufactured utilizing this framework to improve tissue integration and minimize surgical complications [140,141]. Vascular and neural scaffolds employ similar self-expansion features and surface adaptation in order to improve biological performance.

### 7.2. Minimally-Invasive, Deployable Devices

SMPs show promise for deployable applications, such as stents, occlusion devices, and grafts, possessing the ability to recover pre-programmed shapes in the presence of physiological stimuli to allow for compacted device delivery [142]. Examples include bioresorbable SMP stents that expand as the vessel remodels and occlusion devices that expand to seal a site of interest [143].

Soft Robotics and Bio-actuation: In addition to implants and drug carriers, 4D-printing of SMPs supports emerging soft robotics and bio-actuators. SMP actuators could use body temperature or light-activation to drive minimally-invasive surgical tools or microrobots that can perform directed interventions [144]. These devices may have applications in guided drug delivery, endovascular surgery, and rehabilitation, where mechanical compliance is key.

As a whole, these applications highlight an important paradigm of SMP-based 4D-printing in medicine: adaptability, minimally invasively deployment, and dynamic response. While there is potential for this method to foster innovation in the medical field, success in translating this technology to clinical practice requires improvements in biocompatibility, degradation control, sterilization, and packaging.

## 8. Technical Limitations of 4D Printed SMPs

Even though DLP printing has many applications, it is important to address limitations like fatigue resistance, expansion pressure, and shape fixation. Firstly, fatigue resistance, or the ability to resist prolonged stimulus or stress, is important to consider for the long-term reliability of potential applications. Thus, a review by Md Jarir Hossain evaluates possible implications, like cyclic stresses, environmental conditions, surface structures, and unique geometries in 4D systems. Fatigue-resistance testing characterizes the endurance to establish a polymer system’s lifespan. Current findings suggest that there is an inadequate investigation into the impact of printing parameters on fatigue, thus identifying a potential target for future research [145].

Another unique challenge of DLP-printed SMPs is the expansion pressure of the material in response to a certain stimulus, mainly dependent on the viscosity and molecular weight of the resin. For example, in a study by Li et al., high-molecular-weight (high-MW) polymers were shown to improve expansion pressure, but consequently increase viscosity beyond the limitations of DLP printing, requiring dilution that may weaken crosslinking density [146]. This, in turn, reduces structural integrity during shape recovery. Furthermore, issues also arise during printing and curing of the polymer itself. For example, incomplete adhesion between layers, thermal contraction during curing, and variations in the rate of photopolymerization impact the functional performance of SMPs.

Another study by Aldana et al. addresses the implications of shape fixity in fabricating DLP-printed SMP systems. One determining factor is the chemical composition of the photo resin, specifically molecular weight and functionalization. Furthermore, the printing process also determines a system’s shape fixity through crosslinking density, crystalline formation, and post-curing methods. Thus, precise control over shape fixity depends on controlling the interplay between resin composition and processing parameters [122].

### 8.1. Cytotoxicity from Unreacted Monomers and Photoinitiators

One of the critical concerns in the development of SMPs for biomedical applications is the potential cytotoxicity arising from unreacted monomers and residual photoinitiators remaining within the polymer matrix post-fabrication. These residual components can leach out over time, especially in implantable devices intended for prolonged contact with biological tissues, ultimately posing significant risks of inflammatory response and cellular toxicity [17,147]. Additionally, sterilization processes, which are essential for ensuring the safety of biomedical implants, can inadvertently exacerbate these issues. For example, low-temperature plasma sterilization has been reported to alter the surface chemistry of SMPs by increasing oxidation and introducing hydroxyl groups, which may exhibit cytotoxic effects [148]. Similarly, gamma irradiation, while effective for sterilization, can modify critical thermal and mechanical properties of SMPs, such as the *T_g_* and cross-linking density, thereby impacting their shape memory performance and biocompatibility [148]. Balancing these mechanical and biological demands remains a central challenge in SMP design. Recent advancements, including the development of poly(glycerol dodecanoate) acrylate (PGDA)-based SMPs with engineered porosity, have demonstrated improved cytocompatibility, achieving cell viability rates exceeding 90%, while preserving mechanical integrity and appropriate thermal responsiveness for biomedical use [13,149]. These innovations highlight the importance of material selection, processing conditions, and post-fabrication treatments in mitigating cytotoxicity and enhancing the clinical potential of SMP-based devices.

### 8.2. Sterilization Limitations for Printed SMP Devices

Sterilization is a significant hurdle for the clinical translation of SMP devices printed by SLA and DLP. Traditional sterilization methods (e.g., steam autoclaving) are unsuitable due to the effects of heat on the polymers, which can result in material degradation, *T_g_* shifts, and inadequate shape memory performance [150]. Radiation-based methods (e.g., gamma and electron-beam sterilization) may negatively impact polymer networks via chain scission, embrittlement, and discoloration of the recovered shape, which undermines recovery fidelity and mechanical stability [148,151,152]. A summary of sterilization techniques, and possible limitations are summarized in Table 4 [153,154,155,156,157].

There are low-temperature alternatives that are less destructive, but may still prove problematic. Across the board, ethylene oxide (EtO) is useful, but exhibits cytotoxic effects. Incomplete crosslinking may leave residual by-products in the polymer matrix, and hydrogen peroxide plasma can further oxidize the surface, affecting cell compatibility. UV irradiation is an inexpensive surface disinfectant but does not deeply penetrate into all porous SLA/DLP geometries, and excessive exposure to UV can compromise the cross-links [158,159]. All sterilization methods exacerbate the leaching of unreacted monomers from incompletely cured resins, highlighting the need for gentle, non-destructive sterilization schemes that maintain functional integrity and biocompatibility of SMP constructs. A summary of biocompatibility is given in Table 5.

### 8.3. Challenges in Shelf-Life Stability and Packaging of DLP and SLA Printed SMPs

The advent of DLP and SLA has accelerated the fabrication of SMPs for biomedical devices, offering precise control over geometry and tunable mechanical properties for minimally invasive implants, scaffolds, and drug delivery systems. However, clinical translation of these materials extends beyond print fidelity and functional performance to critical issues of storage stability and safe packaging. SMPs must retain predictable shape-memory behavior, mechanical robustness, and biocompatibility throughout manufacturing, sterilization, transportation, and shelf storage prior to implantation. Yet, their chemical sensitivity, environmental vulnerability, and incompatibility with conventional packaging and sterilization workflows present major barriers to regulatory approval and widespread adoption.

SMPs fabricated via DLP and SLA approaches have limited shelf-life due to inherent chemical and physical degradation routes. Residual monomers and photoinitiator species in the photocured network continue to react after fabrication; thus, there is a gradual egress in the *T_g_*, changing modulus, and decline in overall recovery reliability of the shape memory response [148,177]. Hydrolytic degradation in humid conditions and oxidative scission upon exposure to oxygen will expedite molecular segmentation, resulting in embrittlement with diminished recovery strains [140].

Packaging remains a substantial hurdle to the clinical translation of SLA- and DLP-printed SMPs, as it must be able to provide assurance of sterile conditions while minimizing environmental degradation, and compliance with strict medical regulations. While polymeric implants sterilized through conventional methods like gamma irradiation, ethylene oxide, and electron beam exposure exist, these procedures commonly cause some form of molecular damage to photocured SMPs, including chain scission, oxidative crosslinking, and loss of shape memory fidelity [121,178]. Thermal sterilization, such as autoclaving, could be applied, but increased temperatures will relax the programmed shapes and degrade thermomechanical properties [179].

In addition to compatibility of sterilization procedures, packaging must also provide an effective barrier to oxygen, moisture, and UV light [180]. Medical packaging systems that employ multilayer polymeric laminates, aluminum foil composites, and high-barrier fluoropolymer films have been evaluated to extend storage life, but effective use in AM workflows has been limited. Furthermore, the validation of packaging is no small feat; ISO 11607 [162,181] will require some form of performance testing to demonstrate integrity of microbial barrier, mechanical integrity, and sterility maintenance in conditions that simulate transport and shelf storage [181].

New strategies include the use of vacuum-sealed pouches, controlled-atmosphere packaging, and smart packaging with embedded sensors to monitor the humidity or oxidative stress, yielding real-time feedback on device environmental exposure and stability [182,183]. However, these sophisticated systems introduce costs, complexity, and require further investigation into their long-term biocompatibility, especially when considering the clinical sterilization process. Most importantly, the development of SMP formulations that are co-designed with sterilization-compatible packaging will be integral to meeting regulatory approval and ensuring reliable device performance.

Beyond general material and packaging constraints, device-specific challenges must also be addressed. Many SMP-based devices, such as stents and occlusion devices, involve complex geometries and require precise, stimulus-responsive shape recovery. Maintaining these carefully tuned properties during storage, sterilization, and transportation is crucial to device safety and effectiveness.

While these limitations present substantial hurdles, ongoing advancements in SMP chemistry, photopolymer resin formulations, and 4D-printing technologies continue to address these issues. Emerging strategies aimed at enhancing material stability, developing sterilization-compatible packaging, and improving regulatory pathways are expected to significantly improve the shelf life, packaging reliability, and clinical viability of SMP-based medical devices in the near future.

## 9. FDA Regulatory Considerations for 4D Printed Medical Devices

The U.S. Food and Drug Administration (FDA) has offered some technical guidance on additive manufactured devices, notably by releasing their Technical Considerations for Additive Manufactured Devices (2017), which mainly describes AM in the context of traditional 3D-printing [184]. Yet, SLA and DLP as AM processes cannot be construed easily due to differing forms of regulatory challenges designed for 4D-printed implants. Light-based AM platforms, such as SLA or DLP, are attractive tools for the fabrication of high-resolution, patient-specific devices that actively change in vivo in response to stimuli including, but not limited to, heat, pH, or light. Also, the added presence of dynamic actuation for such constructs would distance them from classification as traditional active implantable systems, and pose additional barriers in necessitating the premarket approval (PMA) based regulatory pathway in place of traditional 510(k) clearance [185].

In the case of SLA- and DLP-printed 4D implants, the FDA review process must consider a variety of additional elements beyond the traditional parameters of dimensional accuracy, biocompatibility, and sterilization validation. More specifically, key considerations include: (1) the predictability of stimulus-triggered actuation, (2) the long-term reliability of repeated transformations, and (3) the safety of the stimuli [186,187]. In addition, SLA- and DLP-printed 4D implants are manufactured from photopolymerizable resins, and will therefore warrant further consideration of residual toxicity from photoinitiator residue, long-term stability of post-curing, and batch consistency [188]. Compared to static devices that employ SLA/DLP printing, the regulators will expect to see robust evidence supporting actuation reproducibility under clinically-relevant physiological conditions, and that actuation does not produce unintended tissue interactions [189].

The preclinical testing specifications for 4D SLA/DLP devices utilize the biocompatibility and mechanical verification components of ISO 10993 [122,168,190,191] in addition to characterizing stimulus–response under simulated physiological conditions, fatigue testing of dynamic transformations, and analysis of degradation by-products produced during repeated actuation [192]. Clinical studies must demonstrate not only biocompatibility and functional stability over the implant’s lifespan, but also the predictability of the in vivo shape recovery and its impact on adjacent tissues. Essential follow-up studies would aid in determining whether the SLA/DLP 4D devices maintain functional fidelity, or whether the forms undergo fatigue-related failures long-term.

Lastly, the oversight authority of the FDA encapsulates all aspects of production, as well as post-market surveillance. SLA- and DLP-printed devices will require ISO 13485 [193]-certified quality assurance systems for manufacturing and rigorously validated processes to ensure that actuation can be reproduced. Regulators will also want to control variability associated with photopolymer-based printing by emphasizing resin formulation optimization, curing steps, and establishing dimensional tolerances [150,194]. There will also likely be more intense post-market surveillance responsibilities levied on these devices, such as adverse event monitoring associated with actuation, non-intended shape changes, and device–tissue interactions. Finally, in this lapse of regulatory consensus, it is important that a set of standards for SLA/DLP-based 4D devices, including resin safety, actuation and performance standards, and triggering specifications, are developed by researchers, manufacturers, and regulatory authorities to ensure smooth clinical translation [139]. A summary of SMP regulations is provided in Table 6.

## 10. Conclusions and Future Directions

SMPs have emerged as a promising category of smart biomaterials with programmable shape changes that enable minimally invasive surgery, adaptive implants, and address many challenges associated with regenerative medicine. With their inherent tunability in formulation, hierarchical-printing structure, and multi-stimuli responsiveness, SMPs have enabled innovation in the biomedical field beyond that of rigid and passive polymer systems. SLA- and DLP-printing have elevated SMPs from proof-of-concept to functional biomedical devices. Specifically, SLA is capable of curing photopolymerizable resins with high geometrical complexity, while DLP supports rapid manufacturing via 2D layer-by-layer curing. Together these technologies allow for the design of dynamic microneedles, adaptive scaffolds, dental aligners, and bioresorbable stents with high resolution, flexible designs unlike any previous generations of biomaterials.

Substantial barriers to clinical use include biocompatibility of resin formulations, cytotoxicity of photoinitiators, barriers to the utilization of traditional sterilization procedures, and poor shelf-life. Furthermore, while implementing functional fillers into nanocomposites improves matrix tunability, it does call into question long-term safety. Overall, these obstacles highlight the need for an interdisciplinary solution involving polymer chemistry, medical device engineering, and translational medicine.

The future of SMPs in the biomedical field will be determined by successful implementation of AI as a predictive model, informing formulation optimization through the effective methods of digital twin modeling, as well as sensor embedding to form closed-loop devices that can autonomously adapt to physiological responses. Moreover, prior research discussed must translate laboratory successes to large-scale, manufacturable, and compliant devices through a regulatory pipeline, particularly utilizing innovations to cooperate with global sustainability efforts, such as de-polymerizable supportive structures and recyclable resins. Therefore, SLA and DLP will enable scaling of shape memory polymers to become fundamental materials in personalized medicine, minimally-invasive interventions, and adaptive healthcare.

## Figures and Tables

**Table 2:** SLA/DLP shape-memory resin overview.

Resin Family/ Representative System	Printing Method(s)	*T_trans_*/*T_g_* or *T_m_* (°C)	Shape Fixity (*R_f_*) and Shape Recovery (*R_r_*)	Mechanical Properties	Biocompatibility/Cytotoxicity Findings	References
Thiol–ene/thiol–acrylate SMP networks (e.g., ABA block copolyester, urethane–thiol–ene, TATATO/TMTMP/TCMDA)	SLA, DLP	*T_g_*: 10–50 (dry), <35 (soaked); *T_trans_*: tunable to 36.5	*R_f_* > 95%; *R_r_* near 100%	Tensile: 0.58–1.3 MPa; elongation: 19–38%; stress relax 22–77 min	Antimicrobial; high viability for neural implants; low cytotoxicity	[63,64,65]
Acrylate/methacrylate SMP elastomers (e.g., PGDA-PAA, star-PCL-MA + poly-thiol)	DLP	*T_g_*: −40 to −36	*R_f_*/*R_r_*: tunable via composition	Young’s: 1–24 MPa; tensile: 1.7–7 MPa; elongation: 70–380%	>90% cell viability; suitable scaffolds	[14,66,67]
PCL-based semicrystalline acrylate resins (e.g., PCL-DMA, PCL-triacrylate, PCL-IA, PCL copolymers)	SLA, DLP	*T_g_*: −38 to −40; *T_m_*: crystalline domains	*R_f_* > 90%; programmable speed	Modulus tunable; tensile > 80% conversion; elongation high	Excellent viability; degradable bone scaffolds	[67,68,69,70]
Cyanate-ester-based SMP IPNs and composites (e.g., triple-shape CE networks)	DLP	*T_g_* > 160; processing- dependent	Triple-shape *R_f_* high	High heat distortion; low dielectric; robust	Limited; suitable composites	[71,72]
AELO-based vitrimer SMP resins (e.g., acetoacetylated epoxidized linseed oil + amines)	DLP	*T_g_*: −18; bio-derived	Triple-shape in 30 s at 30 °C	Tensile: 1 MPa; elongation: 31% post-anneal	Bio-sourced; healing for biomedical	[73,74]
PEG-based SMP hydrogels (e.g., PEGDA with thermo-responsive or crystalline segments)	SLA, DLP	*T_g_* tunable; *T_m_* crystalline	*R_f_*/*R_r_* high in hydrogels	Hydrophilic; adjustable modulus	>80% viability; low-immunogenic	[75,76,77]
Polyester-urethane SMP elastomers for DLP (e.g., PGDA-based SME, PU/PCL blends)	DLP	*T_g_*: −38 to −40	*R_f_* tunable via blends	Tunable modulus; high elongation scaffolds	Low cytotoxicity; degradable tissue engineering	[14,68]
Miscellaneous thiol-yne/thiol-X polyester inks for elastic SMP scaffolds	SLA, DLP	*T_g_* variable; degradable	*R_f_* > 95%; tunable	Elastic; high elongation cell-penetrating	Non-immunogenic; low cytotoxicity gene deliver	[78,79,80]
CHI-MA/biobased acrylate hybrids (e.g., chitosan-methacrylate, PLA-PEG)	DLP	*T_g_*/*T_m_* tunable bioink	*R_f_*/*R_r_* tunable	Tunable for hydrogels/ scaffolds	Excellent biocompatibility; anticancer delivery	[77,81,82]

**Table 3:** Biomedical applications summary table.

Application	Material/Type	Printing Technique	Stimulus	Key Advantage	Reference
Intestinal Drug Delivery Systems	poly(β-aminoester) (PBAE) and C18-acrylate	DLP	Temperature, pH, UV irradiation	Environmental stimuli can be utilized to expand and facilitate drug delivery across the GI tract	[103]
Orthodontic Clear Retainers	Dental LT Clear Resin	DLP	Oral environment	Efficient method to directly print patient aligners	[97,98]
Dental Implant Models	1-alkoxylated bisphenol-A dimethacrylate, phosphine oxide, 2-UDMA, diacrylate, acrylic resin, phosphine oxide 3-bis methacrylate, methacrylate monomers, ethyl phenylphosphinate	DLP/SLA	/	High precision implant models with less waste and precise customization using patient scans	[99]
Multi-layered Dentures, Crowns, Bridges, etc.	Objet VeroGlaze MED620 and Objet MED610 Biocompatible Clear	DLP	/	Create more aesthetically pleasing dental restorations	[112]
Microneedles	Wide range of polymer formulations	DLP/SLA	Temperature, pH	High specificity and improved performance of microneedles	[101]
Drug Delivery Systems	PVA-MA-AA	DLP/SLA	pH	Supramolecular interactions can provide a more flexible alternative	[113]
Modified-Release Tablets	poly(ethylene glycol) diacrylate (PEGDA) and poly(ethylene glycol) dimethacrylate (PEGDMA)	DLP	UV	First fabrication of DLP printed solid oral dosage	[102,114]
Peripheral Nervous Tissue Regeneration	GelMA, chitosan, and PEDOT resin	DLP	UV	Very high printing resolution	[115]
Fabrication of Bone Implants	Photopolymerized feedstock derived from human cortical bone	DLP	/	DLP can utilize many different materials specific to desired application	[109,116]
Contact Lenses	methacrylate, diphenyl (2,4,6-trimethylbenzoyl) phosphine oxide	DLP	Temperature	Can utilize different materials and bases to achieve patient specific and efficient printing of contact lenses	[48]
Biodegradable Vascular Stents	PLC and PLA	DLP	/	Can be used to widen blocked blood vessels, while gradually degrading as the vessel heals and the blockage resolves	[111]

**Table 4:** Sterilization methods of SMPs.

Sterilization Method	Temperature	Pros	Cons for 4D-Printed SMPs
Autoclave (Steam)	121–135 °C	Fast, reliable sterilization	Deformation: *T_trans_*/*T_g_* exceeded; Shape-memory effect (SME) loss
Ethylene Oxide (EtO)	<60 °C	Retains shape-memory; low temperature	Moisture triggers premature shape recovery in water-activated systems
Gamma/Electron-Beam (EB)	Ambient	Strong penetration; terminal sterilization	Polymer degradation (chain Scission, crosslinking); SME alteration
Plasma/Hydrogen Peroxide (H_2_O_2_) Vapor	<50 °C	Low-temp, surface sterilization	Limited penetration; potential surface oxidation
Nitrogen Dioxide (NO_2_/ Noxilizer)	25–34 °C	Non-thermal; preserves SME	Experimental stage; lacks broad regulatory validation
Supercritical CO_2_	Ambient	Non-thermal; good penetration	Early-phase; material compatibility and SME data limited
Low-Temperature H_2_O_2_ Vapor	~50 °C	Clinically accepted; low heat	Limited published data on post-sterilization SME recovery

**Table 5:** Biocompatibility and translation readiness matrix.

System/Monomers	Photoinitiator(s)	Short-Term Cytotoxicity (In Vitro, ISO 10993-5) [122]	Long-Term In Vivo Safety	Sterilization Compatibility	Translation Status & Closest to Clinic	Key Data Gaps	References
Thiol-ene (e.g., TATATO/TMTMP, pe ene:acrylate hybrids)	TPO, TPO-L	Low (<10% viability reduction); >70% cell viability in fibroblasts/cortical cultures; minimal unreacted thiols (<1 wt%) due to rapid kinetics	Non-neurotoxic; reduced glial reactivity on softening SMP; no adverse in rodent implants	EtO, low-T H_2_O_2_ vapor, NO_2_ (preserves SME); avoid gamma/EB (crosslinking)	Medium-High; neural conduits, coatings validated ex vivo/small animals	Degradation byproducts (thiols/sulfides) during cyclic actuation; nanofiller leaching	[86,160,161,162,163,164,165]
Acrylate (e.g., PEGDA/PEGDMA, UDMA)	BAPO, TPO	Moderate (10–30% viability loss from leachables > 10 ppm); BAPO more cytotoxic/discoloring than TPO	Limited; potential chronic inflammation from residuals; safe in short-term drug delivery	Plasma/H_2_O_2_, supercritical CO_2_; autoclave damages networks; radiation alters SME	Low-Medium; oral tablets, microneedles in vitro	Long-term monomer hydrolysis products; oxygen inhibition residuals	[8,64,81,86,166,167,168,169,170]
PCL/PLA-based SMP (e.g., PLC/PLA, PCL-DA/PLLA semi-IPN)	TPO, DMPA	Low (ISO 10993 -5) [122]; FDA-cleared analogs; osteoinductive in hMSCs	High biocompatibility; intrinsic osteoinductivity in cranial defects; vascular stents degrading safely	EtO, supercritical CO_2_ preferred; radiation risks crosslinking/acid byproducts	High (closest to clinic); bioresorbable stents/scaffolds in small animals (rats/pigs)	Acidic degradation during repeated actuation (pH drop); shelf-life under fatigue	[14,163,170,171,172,173,174]
PGDA-based (propyl gallate diacrylate, antioxidant SMP)	TPO	Very low (antioxidant quenches radicals; <5% cytotoxicity)	Promising; reduced oxidative stress in scaffolds	Low-temp (H_2_O_2_, NO_2_) preserves SME	High; neural/bone scaffolds in vitro/ex vivo	In vivo long-term (beyond 3 months); actuation-fatigue byproducts	[169,175]
PBAE (poly(β-aminoester), C18-acrylate)	TPO/BAPO	Moderate-short term (pH-sensitive; viable for GI delivery)	Limited; safe in GI tract models	EtO compatible; moisture risks premature response	Medium; intestinal drug systems in vitro	Chronic GI exposure; stimulus crosstalk	[8,81]
GelMA hybrids (GelMA/chitosan/PEDOT, PEGDA)	TPO/DMPA	Low-moderate (macrophage response varies by sterilization); >80% viability	Good neural compatibility; no major inflammation	EtO/EtOH better than autoclave (alters modulus)	Medium; neural tissue regeneration in vitro	Sterilization-induced gene expression changes; long-term resorption	[164,176]

**Table 6:** Comparative regulatory pathway for overview of 3D vs. 4D-Printed devices for SMPs.

Aspects	3D-Printed Implantable SMP Devices	4D-Printed Implantable SMP Devices	References
Definition	Additive manufacturing of SMP-based static implantable biomedical devices	Additively manufactured implantable devices made of SMPs that dynamically change shape over time inside the body post-implantation in response to an external stimulus	[195]
FDA Device Classification	Typically, Class 2 or 3 (510(K) for predicates or PMA for novel devices)	Typically, Class 3 (PMA or De Novo—high scrutiny as active implantable with dynamic in vivo actuation)	[184]
Key Regulatory Concerns	Biocompatibility (ISO 10993 [122,168,190,191] series) Mechanical reliability Sterilization validation 3D printing reproducibility Degradability/product safety	All 3D concerns in addition to: Predictability and repeatability of in vivo actuation Stimulus safety (thermal, pH, light etc.) Long-term actuation reliability and fatigue under cyclic physiological conditions Risk of unintended transformation Potential for dynamic shape recovery affecting surrounding tissue/organs	[189]
Preclinical Study Requirements	ISO 10993 [122,168,190,191] biocompatibility Mechanical and fatigue testing Sterilization and shelf-life studies Degradation kinetics and by-product analysis	All 3D requirements in addition to: Actuation performance testing under simulated physiological conditions Stimulus–response safety and reliability studies	[196]
Clinical Study Requirements	Biocompatibility and safety Mechanical and functional performance over implantation period	All 3D requirements in addition to: Clinical evaluation of in vivo actuation predictability Long-term functional follow-up and monitoring of shape recovery impact on adjacent tissues	[185]
Manufacturing Process Requirements	ISO 13485 [193] certified processes for additive manufacturing, ensuring dimensional accuracy, material consistency and sterility assurance	All 3D requirements in addition to: Process validation for dynamic functionality Actuation reliability checks post-manufacturing	[193]
Regulatory Submission Pathway	510(k) or PMA depending on risk classification and predicate availability	PMA or De Novo application with extensive preclinical and clinical data demonstrating dynamic safety and efficacy	[184,185]
Post-market Surveillance Requirements	Post-market clinical follow-up (PMCF) under MDR Adverse event reporting (MDR/FDA) Device traceability and product recall protocols	Enhanced PMCF including: Monitoring of actuation-related adverse effects Surveillance for unintended shape changes and tissue interactions	[184,194]
Global Regulatory Pathways	FDA (510(k)/PMA) EU MDR (Class IIb/III) TGA (Australia) MDA (Japan) CFDA (China)	Similar pathways as 3D but with added scrutiny under “active implantable” or “high-risk device” categories across global authorities	[142,193,197]
Specific Regulatory Hurdles for 4D printed medical devices	Reproducibility of mechanical properties	All 3D hurdles in addition to: In vivo actuation predictability Dynamic reliability in long-term use Managing stimulus safety and unintended tissue effects	[185]
SMP-specific considerations (3D vs. 4D)	Reproducibility of mechanical properties and shape fixation Degradation by products from hydrolysis (e.g., carboxyl ends in polyesters like PLA/PCL) Biocompatibility of static stimulus exposure (e.g., thermal validation per ISO 10993 [122,168,190,191])	All 3D considerations plus: Actuation reproducibility under physiological cycles (fatigue from repeated shape recovery) Degradation by-products during dynamic actuation (accelerated hydrolysis/oxidation) Stimulus safety (e.g., thermal/pH/light risks to tissues, unintended recovery)	[142,163,185,198]

## Data Availability

No new data were created or analyzed in this study.
